# Ambient Air Pollution and Adult Asthma Incidence in Six European Cohorts (ESCAPE)

**DOI:** 10.1289/ehp.1408206

**Published:** 2015-02-24

**Authors:** Bénédicte Jacquemin, Valérie Siroux, Margaux Sanchez, Anne-Elie Carsin, Tamara Schikowski, Martin Adam, Valeria Bellisario, Anna Buschka, Roberto Bono, Bert Brunekreef, Yutong Cai, Marta Cirach, Françoise Clavel-Chapelon, Christophe Declercq, Roberto de Marco, Audrey de Nazelle, Regina E. Ducret-Stich, Virginia Valeria Ferretti, Margaret W. Gerbase, Rebecca Hardy, Joachim Heinrich, Christer Janson, Deborah Jarvis, Zaina Al Kanaani, Dirk Keidel, Diana Kuh, Nicole Le Moual, Mark J. Nieuwenhuijsen, Alessandro Marcon, Lars Modig, Isabelle Pin, Thierry Rochat, Christian Schindler, Dorothea Sugiri, Morgane Stempfelet, Sofia Temam, Ming-Yi Tsai, Raphaëlle Varraso, Danielle Vienneau, Andrea Vierkötter, Anna L. Hansell, Ursula Krämer, Nicole M. Probst-Hensch, Jordi Sunyer, Nino Künzli, Francine Kauffmann

**Affiliations:** 1 VIMA (Aging and Chronic Diseases, Epidemiological and Public Health Approaches), U1168, INSERM (Institut national de la santé et de la recherche médicale), Villejuif, France; 2 UMR-S 1168, UVSQ (Université Versailles St-Quentin-en-Yvelines), Montigny le Bretonneux, France; 3 Centre for Research in Epidemiology and Population Health (CESP), U1018, Respiratory and Environmental Epidemiology Team, INSERM, Villejuif, France; 4 UMRS 1018, Université Paris Sud, Villejuif, France; 5 CREAL, Centre for Research in Environmental Epidemiology, Barcelona, Spain; 6 IAB, Team of Environmental Epidemiology Applied to Reproduction and Respiratory Health, INSERM, Grenoble, France; 7 IAB, Team of Environmental Epidemiology Applied to Reproduction and Respiratory Health, Université Grenoble Alpes, Grenoble, France; 8 IAB, Team of Environmental Epidemiology Applied to Reproduction and Respiratory Health, CHU de Grenoble, Grenoble, France; 9 Swiss TPH (Swiss Tropical and Public Health Institute), Basel, Switzerland; 10 University of Basel, Basel, Switzerland; 11 IUF, Leibniz Research Institute for Environmental Medicine, Düsseldorf, Germany; 12 Department of Public Health and Pediatrics, University of Turin, Turin, Italy; 13 Institute for Risk Assessment Sciences, University Utrecht, Utrecht, the Netherlands; 14 Julius Center for Health Sciences and Primary Care, University Medical Center Utrecht, Utrecht, the Netherlands; 15 MRC-PHE Centre for Environment and Health, Imperial College London, London, United Kingdom; 16 Nutrition, Hormones and Women Health Team, U1018, Centre for Research in Epidemiology and Population Health (CESP), INSERM, Villejuif, France; 17 InVS, French Institute for Public Health Surveillance, Saint-Maurice, France; 18 Unit of Epidemiology and Medical Statistics, Department of Public Health and Community Medicine, University of Verona, Verona, Italy; 19 Centre for Environmental Policy, Faculty of Natural Sciences, Imperial College London, London, United Kingdom; 20 Section of Biostatistics and Clinical Epidemiology, Department of Public Health, Neuroscience, Experimental and Forensic Medicine, University of Pavia, Pavia, Italy; 21 Division of Pulmonary Medicine, University Hospitals of Geneva, Geneva, Switzerland; 22 MRC Unit for Lifelong Health and Ageing, Research Department of Epidemiology and Public Health, University College, London, United Kingdom; 23 Institute of Epidemiology, German Research Center for Environmental Health (GmbH), Helmholtz Zentrum München, Neuherberg, Germany; 24 Department of Medical Sciences, Respiratory Medicine and Allergology, Uppsala University Hospital, Uppsala, Sweden; 25 Department of Respiratory Epidemiology & Public Health, Imperial College London, London, United Kingdom; 26 Public Health and Clinical Medicine, Umea University, University Hospital, Umea, Sweden; 27 Pédiatrie, CHU de Grenoble, Grenoble France; 28 Directorate of Public Health and Primary Care, Imperial College Healthcare NHS Trust, The Bays, London, United Kingdom; * These authors contributed equally to this work. **Steering Committee ESCAPE Workpackage 4 (Respiratory Health in Adults). ^#^Deceased.

## Abstract

**Background:**

Short-term exposure to air pollution has adverse effects among patients with asthma, but whether long-term exposure to air pollution is a cause of adult-onset asthma is unclear.

**Objective:**

We aimed to investigate the association between air pollution and adult onset asthma.

**Methods:**

Asthma incidence was prospectively assessed in six European cohorts. Exposures studied were annual average concentrations at home addresses for nitrogen oxides assessed for 23,704 participants (including 1,257 incident cases) and particulate matter (PM) assessed for 17,909 participants through ESCAPE land-use regression models and traffic exposure indicators. Meta-analyses of cohort-specific logistic regression on asthma incidence were performed. Models were adjusted for age, sex, overweight, education, and smoking and included city/area within each cohort as a random effect.

**Results:**

In this longitudinal analysis, asthma incidence was positively, but not significantly, associated with all exposure metrics, except for PM_coarse_. Positive associations of borderline significance were observed for nitrogen dioxide [adjusted odds ratio (OR) = 1.10; 95% CI: 0.99, 1.21 per 10 μg/m^3^; *p* = 0.10] and nitrogen oxides (adjusted OR = 1.04; 95% CI: 0.99, 1.08 per 20 μg/m^3^; *p* = 0.08). Nonsignificant positive associations were estimated for PM_10_ (adjusted OR = 1.04; 95% CI: 0.88, 1.23 per 10 μg/m^3^), PM_2.5_ (adjusted OR = 1.04; 95% CI: 0.88, 1.23 per 5 μg/m^3^), PM_2.5absorbance_ (adjusted OR = 1.06; 95% CI: 0.95, 1.19 per 10^–5^/m), traffic load (adjusted OR = 1.10; 95% CI: 0.93, 1.30 per 4 million vehicles × meters/day on major roads in a 100-m buffer), and traffic intensity (adjusted OR = 1.10; 95% CI: 0.93, 1.30 per 5,000 vehicles/day on the nearest road). A nonsignificant negative association was estimated for PM_coarse_ (adjusted OR = 0.98; 95% CI: 0.87, 1.14 per 5 μg/m^3^).

**Conclusions:**

Results suggest a deleterious effect of ambient air pollution on asthma incidence in adults. Further research with improved personal-level exposure assessment (vs. residential exposure assessment only) and phenotypic characterization is needed.

**Citation:**

Jacquemin B, Siroux V, Sanchez M, Carsin AE, Schikowski T, Adam M, Bellisario V, Buschka A, Bono R, Brunekreef B, Cai Y, Cirach M, Clavel-Chapelon F, Declercq C, de Marco R, de Nazelle A, Ducret-Stich RE, Ferretti VV, Gerbase MW, Hardy R, Heinrich J, Janson C, Jarvis D, Al Kanaani Z, Keidel D, Kuh D, Le Moual N, Nieuwenhuijsen MJ, Marcon A, Modig L, Pin I, Rochat T, Schindler C, Sugiri D, Stempfelet M, Temam S, Tsai MY, Varraso R, Vienneau D, Vierkötter A, Hansell AL, Krämer U, Probst-Hensch NM, Sunyer J, Künzli N, Kauffmann F. 2015. Ambient air pollution and adult asthma incidence in six European cohorts (ESCAPE). Environ Health Perspect 123:613–621; http://dx.doi.org/10.1289/ehp.1408206

## Introduction

Asthma has a high prevalence of 5–10% (Eder et al. 2006), and in 2010 ranked as the 28th leading cause of disability-adjusted life years worldwide (Murray et al. 2012). Asthma is a heterogeneous disease that may appear at any age (most often in childhood), and can persist, possibly remit, or show variable activity over time (Strachan et al. 1996; Wenzel 2012). The complexity of this chronic disease is particularly challenging, and more research is needed on the environmental determinants of the disease (and not only on the acute triggers of attacks), because the increase in asthma incidence over the last decades (Eder et al. 2006) strongly suggests a role of environmental factors. The role of air pollutants in triggering asthma exacerbations in young and adult asthma patients is established (Peel et al. 2005; Sunyer et al. 1997). Several studies support the role of air pollution in the development of asthma in childhood (Anderson et al. 2013; McConnell et al. 2010), but not all (Mölter et al. 2015). The role of air pollution in adult-onset asthma (i.e., asthma incidence) has been investigated in only a few studies (Anderson et al. 2013; Jacquemin et al. 2012; Young et al. 2014) and should not be extrapolated from studies in children because childhood-onset and adult-onset asthma are two distinct asthma phenotypes that have, at least partly, different clinical, biological, and genetic characteristics (Wenzel 2012). Among studies in adults, only four have used individually assigned air pollution estimates at home addresses. A small Swedish case–control study (203 cases and 203 controls) suggested an association of traffic-related nitrogen dioxide (NO_2_) with asthma incidence, but the study lacked statistical power (Modig et al. 2006). Both the Respiratory Health in Northern Europe (RHINE) study (3,824 participants) (Modig et al. 2009) and the European Community Respiratory Health Survey (ECRHS) (4,185 participants) (Jacquemin et al. 2009b) reported a positive association between NO_2_ and asthma incidence. The Swiss Study on Air Pollution and Health in Adults (SAPALDIA) found similar results, but only in never-smokers and using source-specific models of local traffic-related particulate matter (PM) as a marker of exposure (Künzli et al. 2009). A recent U.S. study suggested an association of PM_2.5_ (≤ 2.5 μm) with incident asthma in women (Young et al. 2014). Two recent reviews concluded that the existing evidence suggests a possible role of air pollution in adult-onset asthma but that the evidence is not conclusive because the studies lacked of power, suggesting the need for larger cohorts (Anderson et al. 2013; Jacquemin et al. 2012).

The European Study of Cohorts for Air Pollution Effects (ESCAPE) developed, for the first time at large scale, fully standardized air pollution measurement, modeling, and assignment methods to individually characterize home outdoor exposure (Beelen et al. 2013; Eeftens et al. 2012). We took advantage of a follow-up of > 10 years among 23,704 adults in six prospective cohorts from eight countries to assess the association between long-term exposure to ambient air pollution and asthma incidence in adulthood.

## Methods

*Study population and assessment of asthma incidence*. Six prospective cohorts from 24 areas in eight countries contributed to the analysis of asthma incidence in adulthood over a 10-year period. Three of these cohorts [ECRHS (ECRHS II Steering Committee 2002), the French Epidemiological study on the Genetics and Environment of Asthma (EGEA) (Siroux et al. 2009), and SAPALDIA (Ackermann-Liebrich et al. 2005)] were respiratory epidemiological cohorts, with detailed information regarding respiratory symptoms, bronchial challenge tests, and sensitization. The three others were general health cohorts. The study on the influence of Air pollution on Lung function, Inflammation and Aging (SALIA; Schikowski et al. 2010) and SAPALDIA were originally designed to investigate effects of air pollution. ECRHS, SAPALDIA, and the Medical Research Council’s National Survey of Health and Development (NSHD) (Kuh et al. 2011) corresponded to a representative sample of subjects of predefined areas. The Etude Epidémiologique auprès de femmes de la Mutuelle Générale de l’Education Nationale (E3N) (Clavel-Chapelon et al. 1997) and SALIA were conducted in elderly women. EGEA included by design a high proportion of relatives of asthma patients recruited in chest clinics. ECRHS, EGEA, and SAPALDIA were initiated in the 1990s and followed-up 9–12 years later. NSHD is a birth cohort of participants born in 1946 and with > 20 regular follow-ups since then; for this analysis, baseline was considered in 1989 and follow-up in 1999. E3N women were recruited in 1990 and followed-up every 2 years; the last follow-up included for this analysis is the one from 2008. SALIA women were recruited in 1985; a questionnaire follow-up was conducted in 2006 and a second from 2007 to 2010. For detailed information on each study, see Supplemental Material, Table S1 and Figure S1.

For each cohort, the absence of asthma at baseline and the incidence of asthma during follow-up were defined as shown in Supplemental Material, Table S2, according to the availability of each cohort’s variables. Two principles were followed regarding the assessment of asthma: harmonization across cohorts and optimal use of available information. Depending on the cohort, asthma was defined by two standardized questionnaires: the British Medical Research Council questionnaire (Samet 1978), which originated in the 1960s, and the ECRHS questionnaire (Burney et al. 1994), designed in the 1990s. For all studies, asthma incidence was defined only in subjects without asthma at baseline. To further improve the specificity of our asthma incidence definition (Pekkanen et al. 2005; Sunyer et al. 2007), we also excluded from the population at risk of new-onset asthma any participant who reported at baseline three of five asthma-like symptoms in the preceding 12 months (wheeze and breathlessness; chest tightness; attack of shortness of breath at rest; attack of shortness of breath after exercise; awakening by attack of shortness of breath); this information was available in three of the six cohorts (ECRHS, EGEA, SAPALDIA) (Boudier et al. 2013). (For flow charts and criteria used to classify asthma for each cohort, see Supplemental Material, Table S2 and Figure S1.) In ECRHS, SAPALDIA, and EGEA, objective asthma-related traits were available. Methacholine bronchial provocation tests were performed and bronchial responsiveness defined when the provocative dose to decrease by 20% the forced expiratory flow volume in 1 sec was ≤ 1 mg cumulative dose of methacholine. Allergic sensitization was assessed as at least one skin prick test or at least one specific immunoglobulin E > 0.35 U/mL (see Table 1 for details). In all studies, hay fever was recorded by questionnaire at baseline and follow-up. Eczema was assessed in some studies. Moving status was defined based on the available data, considering addresses (geocodes) when baseline address was available and reported move assessed through questionnaire otherwise. Ethical approval was obtained for each cohort/center from the appropriate institutional or regional ethics committee, and written consent was obtained from each participant.

**Table 1:** Characteristics of participants with NO_2_ exposure estimates in the ESCAPE analyses, by study (*n*) and outcome.

Characteristic	All (23,704)		ECRHS (3,802)		EGEA (517)^*a*^		E3N (12,763)		NSHD (2,339)		SALIA (2,073)		SAPALDIA (2,210)	
	No asthma	Incident asthma	No asthma	Incident asthma	No asthma	Incident asthma	No asthma	Incident asthma	No asthma	Incident asthma	No asthma	Incident asthma	No asthma	Incident asthma
*n*	22,447	1,257	3,657	145	468	49	12,012	751	2,245	94	1,925	148	2,140	70
Female (%)	82	89	52	67*	54	57	100	100	52	60	100	100	53	61
Age at baseline (years) (mean ± SD)	42	46	34 ± 7	34 ± 7	41 ± 12	36 ± 13*	49 ± 7	49 ± 6*	43 ± 0	43 ± 0	54 ± 1	55 ± 1	42 ± 12	38 ± 11*
Age ≥ 50 at baseline (%)	35	36	0	0	23	14*	43	38*	0	0	100	100	31	16*
Age at follow-up (years) (mean ± SD)	60	60	43 ± 7	42 ± 7	52 ± 12	47 ± 13*	65 ± 7	64 ± 6	53 ± 0	53 ± 0	71 ± 3	72 ± 3*	53 ± 12	49 ± 11
BMI at baseline (kg/m^2^) (mean ± SD)	23	24	24 ± 4	24 ± 5	23 ± 3	23 ± 5	22 ± 3	23 ± 3*	25 ± 4	27 ± 5*	27 ± 4	27 ± 4	24 ± 4	24 ± 4
BMI ≥ 25 at baseline (%)	26	30	33	35	26	31	13	18*	45	67*	67	66	31	29
Smoking status at baseline (%)
Current smoker	22	22	36	30	25	47*	17	19	26	29	11	16	36	24
Ever-smoker	29	29	21	26	24	10*	34	34	42	39	9	5	21	27
Never-smoker	49	49	43	45	51	43*	50	46	31	32	80	79	43	49
Maximum education at baseline or follow-up (%)
Low level	12	13	23	28	26	17	2	3*	41	51	22	25	7	9
Medium level	24	21	34	28	22	15	6	8*	48	42	49	51	62	60
High level	64	66	43	45	52	67	91	88*	11	8	29	24	30	31
Movers (between baseline and follow-up) (%)	33	33	45	42	45	55	27	31	39	37	18	15	48	50
Asthma-related variables
Methacholine test,^*b*^ baseline (*n*)	4,837	197	2,871	112	385	38	NA	NA	NA	NA	NA	NA	1,581	47
PD20 ≤ 1 mg (%)	9	28	8	38*	12	29*	NA	NA	NA	NA	NA	NA	9	6
Methacholine test,^*b*^ follow-up (*n*)	3,499	147	2,197	94	264	25	NA	NA	NA	NA	NA	NA	1,038	28
PD20 ≤ 1mg (%)	9	40	10	44*	12	48*	NA	NA	NA	NA	NA	NA	5	18*
SPT/spIgE,^*c*^ baseline (*n*)	5,207	228	2,937	119	457	49	NA	NA	NA	NA	NA	NA	1,813	60
Allergic sensitization (%)	27	50	25	52*	35	45	NA	NA	NA	NA	NA	NA	28	50*
SPT/spIgE,^*c*^ follow-up (*n*)	4,684	194	2,859	112	371	40	NA	NA	NA	NA	NA	NA	1,454	42
Allergic sensitization (%)	27	55	24	55*	33	55*	NA	NA	NA	NA	NA	NA	30	55*
Hay fever at baseline (%)	13	26	19	46*	25	35	11	25*	16	19	5	10*	17	40*
Hay fever at follow-up (%)	11	27	21	54*	29	64*	5	17*	23	40*	5	19*	18	51*
Eczema at baseline (%)	34	43	33	43*	23	29	NA	NA	NA	NA	NA	NA	38	51*
Eczema at follow-up (%)	27	36	35	48*	25	37	NA	NA	NA	NA	4	13*	35	57*
Abbreviations: BMI, body mass index; NA, not available; PD20, dose of methacholine required to produce a 20% fall in the forced expiratory volume in 1 sec; SPT/splgE, skin prick test/specific immunoglobulin E. Percentages are column percentages. ^***a***^In EGEA, the 517 participants belong to 372 families, and 24% of the participants had at least one parent with asthma. ^***b***^Bronchial hyperresponsiveness was defined dichotomously as PD20 ≤ 1 mg [the common dose used in all three studies (ECRHS, EGEA, and SAPALDIA)]. ^***c***^Allergic sensitization at baseline and follow-up for ECRHS, EGEA, and SAPALDIA was defined as at least one skin prick test (SPT) positive or at least one specific immunoglobulin E > 0.35 U/mL. In ECRHS, allergic sensitization was defined at baseline as any positive SPT (7 allergens tested) and allergic sensitization at follow-up was defined as any specific IgE concentration > 0.35 U/mL (4 IgEs tested). In EGEA and SAPALDIA, allergic sensitization at baseline or follow-up was defined as any positive SPT (EGEA: 11 and 12 allergens tested at baseline and follow-up, respectively; SAPALDIA: 8 allergens tested). *p < 0.05 comparing cohort participants with and without incident asthma.														

The covariates were chosen based on evidence from previous studies (Jacquemin et al. 2009b; Künzli et al. 2009; Modig et al. 2009) but also taking into account the assessment and quality of available data within the ESCAPE cohorts. Smoking (current, former, never), maximum educational level (low, medium, high), and overweight [body mass index (BMI) < 25, ≥ 25 kg/m^2^, except in ECRHS where an additional missing category was created because > 20% of data were missing for this variable] were considered in the analysis.

City/area refers to the city in ECRHS, EGEA, E3N, and SAPALDIA and the country in NSHD (England, Wales, and Scotland). All SALIA participants came from one area.

*Exposure data*. Measurements of NO_2_/NO_x_ (nitrogen oxides) were conducted in three seasons in 2010 or 2011 using passive samplers in the 24 areas. Areas refers to cities (with or without their metropolitan areas) in most of the cases, except in the United Kingdom where it is the whole country and in the Ruhr region in Germany where it is an urban area including several cities. PM monitoring campaigns were conducted in 12 areas. Exposure estimates at the participants’ addresses at follow-up [NO_2_, NO_x_, PM_10_ (≤ 10 μm), PM_2.5_, PM_2.5absorbance_, PM_coarse_] derived from land use regression (LUR) models were used as primary exposure covariates (Beelen et al. 2014; Eeftens et al. 2012; see also http://www.escapeproject.eu). Back-extrapolated exposure estimates for NO_2_ and PM_10_ were used for sensitivity analyses because ESCAPE air pollution measurement campaigns took place after the health surveys for most cohorts. The back-extrapolated concentration was estimated by multiplying the modeled ESCAPE annual mean concentration by the ratio between average annual concentrations as derived from the routine monitoring site(s) for the period in the past and for the ESCAPE measurement period time (Beelen et al. 2014). Exposures were back-extrapolated to the follow-up period using routinely available air pollution monitoring data, but could not be extrapolated to baseline for all the areas because of a lack of earlier monitoring data for some cities, particularly for PM_10_. Furthermore, baseline addresses were not available in all the cohorts. Traffic exposure indicators, traffic intensity (on the nearest road), and traffic load (in a 100-m buffer) were derived from geographic databases.

*Data analysis*. The following cohort-specific random-effects logistic regressions were performed for all air pollution metrics: unadjusted (model 1), adjusted for age and sex (model 2), and additionally adjusted for smoking, overweight, and education level at baseline (model 3, the main model). Cox regression analysis was not used due to imprecision of the date of onset. The heterogeneity of the effect estimates between the cohorts was tested using the chi-square test. Meta-analytic estimates were estimated using fixed-effects models in the absence of heterogeneity between cohorts (*p*-value of heterogeneity > 0.1), and using random-effects models when heterogeneity between cohorts was present. The *I*^2^ statistic was calculated for quantifying heterogeneity. For meta-analyses of subgroups (age, sex, and smoking status), meta-analytic stratum-specific estimates were derived and were compared between strata. Cohort-specific estimates in subgroup analyses were conducted using model 3, but without taking into account random effects per city/area because random-effects models encountered convergence problems.

Because NO_2_ is not measured near busy roads, models of associations with traffic variables were adjusted for background NO_2_. Random effects were used for the main relevant cluster for each cohort (city/area for E3N, ECRHS, NSHD, and SAPALDIA, or family for EGEA).

Sensitivity analyses were conducted *a*) to address the robustness of the association to a change in the window of exposure (by using back-extrapolated NO_2_ and PM_10_); *b*) to address the possible impact of the exposure models’ performance [by restricting analyses to areas where exposure models had the highest predictive value (cross-validation *R*^2^ > 0.6)]; *c*) to better compare the NO_2_ with the PM results (by restricting NO_2_ analyses to participants who also had PM measurements); *d*) to unmask a possible effect of one pollutant over the other using a two-pollutant model (NO_2_ and PM_10_); *e*) excluding individuals with a self-reported age-at-onset ≥ 2 years prior baseline according to record at follow-up, to better capture adult-onset asthma and not reappearance of childhood onset of asthma (Jacquemin et al. 2009b); this analysis is referred to as incidence with coherent age of onset in tables; *f*) excluding individuals with exposures at both upper and lower 5% extremes of pollutant values; and *g*) adjusting for “study city/area” as a fixed effect instead of random effect, as used before (Jacquemin et al. 2009b) but debated (Neuhaus and Kalbfleisch 1998). Stratified analyses were conducted by age (< 50 or ≥ 50 years), sex, and smoking (ever- or never-smokers) and analyses restricted to nonmovers were conducted. We investigated the robustness of the meta-analyses estimates by excluding consecutively each cohort. We performed further analyses within the ECRHS cohort to allow direct comparison with a previous ECRHS publication (Jacquemin et al. 2009b) that estimated NO_2_ using the APMoSPHERE (Air Pollution Modelling for Support to Policy on Health and Environmental Risk in Europe) model, a 1 × 1 km surface model developed using GIS-based techniques (Vienneau et al. 2009).

All the results are shown for an increase of 10 μg/m^3^ of NO_2_ and PM_10_, 5 μg/m^3^ of PM_2.5_ and PM_coarse_, 10^–5^/m^1^ of PM_2.5absorbance_ and 20 μg/m^3^ of NO_x_. For traffic measures, the results are shown for an increase of 5,000 vehicles/day for traffic intensity on the nearest road and four millions vehicles × m/day for traffic load in major roads within a 100-m buffer. Analyses used Stata version 12 (StataCorp, College Station, TX, USA).

## Results

*Population*. The six cohorts contributed to 1,257 incident cases of asthma for the total population of 23,704 participants (Table 1). Cohorts differed by several characteristics, reflecting recruitment differences. Asthma incidence rates varied from 2.9/1,000/year in SAPALDIA to 8.3/1,000/year in EGEA. In the three cohorts (ECRHS, EGEA, SAPALDIA) with available data, participants who developed asthma after baseline (i.e., incident asthma cases) were more likely than other participants to be classified as having bronchial hyperresponsiveness (BHR) at baseline (28% vs. 9% with a positive methacholine test), and were even more likely to have BHR at follow-up (40% compared with 9%). Compared with subjects who did not develop asthma, those with incident asthma exhibited more allergic sensitization, before (baseline) and after (follow-up) the onset of asthma. Hay fever was twice as common among participants with incident asthma compared with those without asthma (except for NSHD and EGEA at baseline).

**Figure 1:**
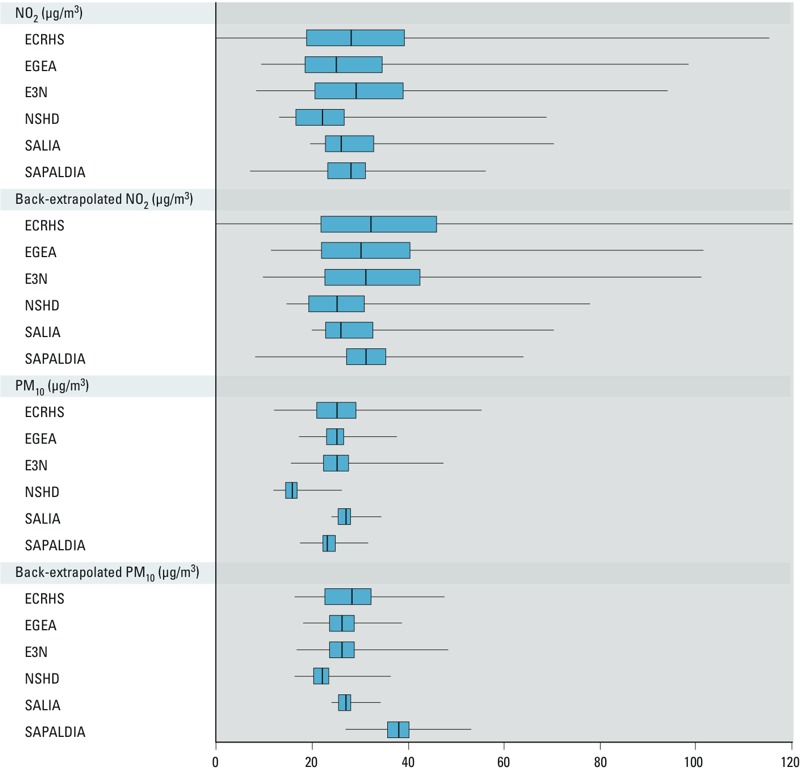
NO_2_ and PM_10_ concentrations (μg/m^3^) by study. Boxes extend from the 25th to the 75th percentile, bars inside the boxes represent the median, and whiskers indicate the minimum and maximum values.

*Air pollution and traffic metrics*. Mean and median air pollution exposures were lower for the NSHD cohort compared with the other five cohorts, though distributions overlapped among the cohorts (Figure 1; see also Supplemental Material, Table S3). The highest mean NO_2_ concentration was found in E3N (31 ± 13 μg/m^3^) and the lowest in NSHD (22 ± 7 μg/m^3^). For PM_10_, the highest mean concentration was found in SALIA (27 ± 2 μg/m^3^) and the lowest in NSHD (16 ± 2 μg/m^3^). Cohort-specific interquartile ranges (IQRs) indicated substantial variability in the exposure contrasts within cohorts, ranging from 8 to 20 μg/m^3^ and 2 to 8 μg/m^3^ for NO_2_ and PM_10_, respectively (see Supplemental Material, Table S3). The highest correlation coefficients were always seen between NO_2_ and NO_x_ (*r* > 0.90) (see Supplemental Material, Table S4). Correlation coefficients between NO_2_ and PM_10_ varied from 0.53 in E3N to 0.83 in SAPALDIA. Correlation coefficients between the different air pollutant concentrations and the traffic indicators showed wide between-cohort heterogeneity (from 0.06 for NO_2_ and traffic intensity in NSHD to 0.81 for PM_2.5absorbance_ and traffic load within a 100-m buffer in EGEA) (Table S4). All the LUR models had a leave-one-out cross validation *R*^2^ > 50%, and most of them > 80% (Beelen et al. 2013; Eeftens et al. 2012) (see Supplemental Material, Table S5).

**Figure 2:**
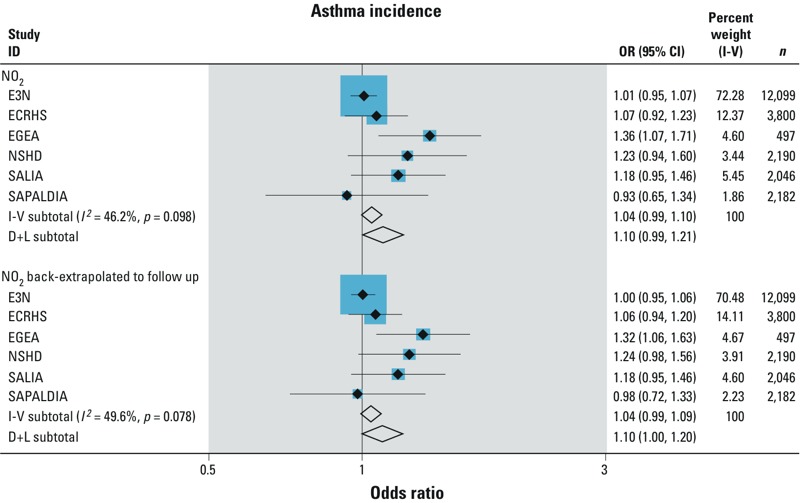
Associations of NO_2_ and NO_2_ back-extrapolated (per 10 μg/m^3^) on asthma incidence. Meta-analysis from the study-specific adjusted random-effects logistic regression models. The logistic regression models were adjusted for age, sex, smoking, overweight, and education level (model 3) with random effects per city/area for each study except for SALIA, where there is only one area, and EGEA, where family structure was taken into account. I-V: inverse variance weighted (fixed effect) pooled estimate of all studies. *I*^2^: variation in estimate effect attributable to heterogeneity. D+L: DerSimonian and Laird (random effect) pooled estimate of all studies. Study-specific odds ratios are shown as solid black diamonds with horizontal lines representing 95% CIs. The size of the blue squares reflects the statistical weight of the study in the meta-analyses. The meta-analytic odds ratios are shown as open black diamonds, the middle of the diamond corresponds to the odds ratio value, and the width of the diamond represents the 95% CI.

*Associations between air pollutants and traffic metrics and asthma incidence*. The unadjusted, simple (adjusted by sex and age), and fully adjusted models provided similar results in individual cohorts (Table 2). The fully adjusted meta-analytic estimate for NO_2_ was positive [odds ratio (OR) = 1.10; 95% confidence interval (CI): 0.99, 1.21; *p* = 0.10]. The association did not change when using the back-extrapolated NO_2_ ESCAPE estimates (OR = 1.10; 95% CI: 1.00, 1.20) (Table 2 and Figure 2). When adjusting by city/area as a fixed effect (instead of random effect), the OR for NO_2_ increased to 1.14 (95% CI: 1.01, 1.29) (Table 3), changes being driven mainly by an increased association estimate in ECRHS (OR = 1.41; 95% CI: 1.10, 1.80 instead of OR = 1.07; 95% CI: 0.92, 1.23).

**Table 2:** Meta-analyses of associations between air pollutants and traffic indicators and the risk for asthma incidence.

Exposure	Increase	OR (95% CI)			Heterogeneity between cohorts (model 3)	
		Model 1	Model 2	Model 3	*I*^2^**(%)	*p*-Value
NO_x_, no. of participants		23,693	23,693	22,814
NO_2_	10 μg/m^3^	1.11 (1.00,1.23)	1.04 (0.99,1.09)	1.10 (0.99,1.21)	46.2	0.10
NO_2_ back-extrapolated to follow-up	10 μg/m^3^	1.10 (1.00,1.21)	1.04 (0.99,1.09)	1.10 (1.00,1.20)	49.6	0.08
NO_x_	20 μg/m^3^	1.09 (1.00,1.18)	1.04 (0.99,1.08)	1.04 (0.99,1.08)	39.8	0.14
PM, no. of participants		17,798^*b*^	17,798^*b*^	17,098^*b*^
PM_10_	10 μg/m^3^	1.05 (0.89,1.24)	1.05 (0.89,1.24)	1.04 (0.88,1.23)	0.0	0.44
PM_10_ back-extrapolated to follow-up	10 μg/m^3^	1.04 (0.88,1.24)	1.04 (0.88,1.24)	1.04 (0.87,1.24)	0.0	0.78
PM_coarse_	5 μg/m^3^	0.98 (0.86,1.12)	0.98 (0.86,1.12)	0.99 (0.87,1.14)	0.0	0.61
PM_2.5_	5 μg/m^3^	1.11 (0.80,1.54)	1.04 (0.88,1.23)	1.04 (0.88,1.23)	24.2	0.25
PM_2.5absorbance_	10^–5^/m	1.05 (0.94,1.16)	1.05 (0.94,1.17)	1.06 (0.95,1.19)	44.5	0.11
Traffic variables, no. of participants^*a*^		22,430	22,428	21,551
Traffic intensity on nearest road	5,000 vehicles/day	1.06 (0.98,1.14)	1.05 (0.98,1.13)	1.05 (0.98,1.13)	56.4	0.04
Traffic load in a 100-m buffer	4,000,000 vehicles × m/day	1.11 (0.94,1.31)	1.09 (0.94,1.27)	1.10 (0.93,1.30)	57.4	0.04
Model 1: unadjusted; model 2: adjusted for age and sex; model 3: adjusted for age, sex, smoking, overweight, and education level. The logistic regression models were conducted with random effects per city/area for each study except for SALIA, where there was only one area, and EGEA, where family structure was taken into account. The OR corresponds to the fixed effect when the *p*-value for heterogeneity was > 0.1; when the *p*-value for heterogeneity was < 0.1, the random effect is stated. *I*^2^: variation of estimate effect attributable to heterogeneity. ^***a***^For traffic intensity on the nearest road. ^***b***^For PM_10_.						

**Table 3:** Results from random-effects meta-analyses for adjusted association between asthma incidence per 10-μg/m^3^ increase for NO_2_ and PM_10_: sensitivity and stratified analyses.

Analysis	No. of subjects		OR (95% CI) from model 3^*a*^		Heterogeneity between cohorts			
	NO_2_	PM_10_	NO_2_	PM_10_	*I*^2^ NO_2_ (%)	*p*-Value	*I*^2^ PM_10_ (%)	*p*-Value
Main analyses	22,814	17,098	1.10 (0.99,1.21)	1.04 (0.88,1.23)	46.20	0.10	0.00	0.44
Stratified analyses
By age*
Restricted to age < 50	14,875	10,499	1.08 (0.96,1.21)	1.07 (0.86,1.32)	56.60	0.06	10.60	0.35
Restricted to age ≥ 50	7,909	6,287	1.02 (0.94,1.12)	1.05 (0.78,1.42)	0.00	0.54	0.00	0.72
By sex**
Men only	4,098	2,264	1.06 (0.92,1.24)	1.00 (0.63,1.59)	0.00	0.45	0.00	0.61
Women only	18,725	14,751	1.07 (0.97,1.19)	1.07 (0.91,1.26)	0.45	0.11	0.00	0.51
By smoking status^#^
Ever-smokers only	11,664^*b*^	8,576	1.13 (0.99,1.29)	1.17 (0.79,1.74)	49.80	0.08	40.30	0.14
Never-smokers only	11,159^*b*^	8,433	1.01 (0.88,1.16)	1.10 (0.87,1.39)	50.00	0.08	0.00	0.52
Sensitivity analyses
Using asthma incidence definition with coherent age of onset (NSHD excluded)	19,935	14,585	1.09 (0.93,1.28)	1.07 (0.59,1.93)	65.40	0.02	64.10	0.03
Among nonmovers	15,289	11,780	1.04 (0.98,1.11)	1.12 (0.91,1.37)	0.00	0.50	0.00	0.88
Excluding E3N	10,715	7,185	1.15 (1.03,1.27)	1.17 (0.82,1.66)	11.90	0.34	8.30	0.36
Excluding ECRHS	19,014	15,151	1.12 (0.98,1.29)	1.13 (0.83,1.55)	56.50	0.06	15.40	0.32
Excluding EGEA	22,317	16,790	1.03 (0.98,1.09)	1.02 (0.86,1.20)	3.50	0.39	0.00	0.79
Excluding NSHD	20,624	15,121	1.08 (0.98,1.20)	1.06 (0.84,1.32)	49.10	0.10	13.80	0.33
Excluding SALIA	20,768	15,052	1.09 (0.98,1.21)	1.03 (0.85,1.26)	50.10	0.09	5.90	0.37
Excluding SAPALDIA	20,632	16,191	1.11 (1.00,1.24)	1.05 (0.89,1.24)	55.20	0.06	0.00	0.42
Excluding 5% upper and lower extreme values	20,642	15,412	1.03 (0.97,1.10)	1.11 (0.89,1.37)	0.00	0.92	0.00	0.84
Fixed effects between cities/areas within the same study	22,814	17,098	1.14 (1.01,1.29)	1.05 (0.86,1.29)	59.20	0.03	2.00	0.40
Restricted to cities/areas with both NO_2_ and PM_10_	17,097^*c*^	17,097^*c*^	1.11 (0.99,1.24)	1.04 (0.88,1.23)	39.40	0.14	0.00	0.44
Restricted to cities/areas with high goodness of fit for NO_2_ exposure models (*R*^2^ ≥ 0.6)	21,048	NA	1.09 (0.98,1.21)	NA	47.40	0.09	NA	NA
Two-pollutant model (NO_2_, PM_10_)	17,097	17,097	1.17 (0.99,1.38)	0.98 (0.79,1.21)	46.20	0.10	0.00	0.42
NA, not applicable. ^***a***^Meta-analysis from the study-specific adjusted logistic regression models. The logistic regression models were adjusted for age (except for the model stratified by age), sex (except for the model stratified by sex), smoking (except for the model stratified by smoking), overweight, and education level (model 3). Random effects are given per city/area (except for the model considering city/area as fixed effect) for each study except for SALIA, where there is only one area, and EGEA, where family structure was taken into account. ^***b***^Inconsistent *n* due to NSHD: 11,664 + 11,159 = 22,823 ≠ 22,814. ^***c***^Inconsistent *n* due to ECRHS. *p-Value for interaction between participants < 50 and ≥ 50 years old for NO_2_: 0.88 and for PM_10_: 0.99. **p-Value for interaction between males and females for NO_2_: 0.66 and for PM_10_: 0.80. ^#^*p*-Value for interaction between smokers and nonsmokers for NO_2_: 0.35 and for PM_10_: 0.69.								

NO_2_ estimates were positive in all sensitivity and stratified analyses (Table 3). Using the stricter definition of asthma incidence with coherent age of onset did not modify the associations but confidence intervals were wider as power was decreased (Table 3). The analyses that were restricted to nonmovers or that excluded the 5% extreme value of the pollutants tended to decrease the associations (OR = 1.04; 95% CI: 0.98, 1.10 and OR = 1.03; 95% CI: 0.97, 1.10; respectively) (Table 3). Although cohort-specific association estimates suggested the possibility of between-cohort differences, with stronger estimates in the French EGEA cohort compared with the others (Figure 2), heterogeneity among the cohorts was not statistically significant (Table 2). After consecutive exclusion of each cohort in the meta-analyses, the point estimate of the OR always remained positive, varying from 1.03 to 1.15, reaching significance for NO_2_ after the exclusion of E3N (OR = 1.15; 95% CI: 1.03, 1.27 with a decreased heterogeneity between cohorts’ estimates) (Table 3) or SAPALDIA (OR = 1.11; 95% CI: 1.00, 1.24). A trend for stronger association between NO_2_ and asthma incidence was observed in ever-smokers compared with never-smokers (OR = 1.13; 95% CI: 0.99, 1.29 and OR = 1.01; 95% CI: 0.88, 1.16, respectively) (*p*-interaction = 0.35) (Table 3). Neither age nor sex modified the associations between NO_2_ and asthma incidence (*p*-interaction = 0.88 and 0.66, respectively) (Table 3). Restricting the analyses either to centers with both NO_2_ and PM_10_ measurements or to areas with a high goodness of fit of the LUR models did not modify the associations between NO_2_ and asthma incidence.

For PM_10_, meta-estimates were similar and not significant in models with or without back-extrapolation (OR = 1.04; 95% CI: 0.87, 1.24 and OR = 1.04; 95% CI: 0.88, 1.23, respectively) (Table 2). Except for PM_coarse_, estimates were all positive but not significant, though borderline significant for NO_x_ (OR = 1.04; 95% CI: 0.99, 1.08) (Table 2).

PM_10_ estimates were positive and tended to increase in any sensitivity analysis, except when excluding EGEA, but never reached significance (Table 3). The analyses that were restricted to nonmovers or excluded the 5% extreme value of the pollutants tended to increase the associations (OR = 1.12; 95% CI: 0.91, 1.37 and OR = 1.11; 95% CI: 0.89, 1.37, respectively). In the stratified analyses, slightly stronger associations between PM_10_ and asthma incidence were observed in ever-smokers compared with never-smokers (OR = 1.17; 95% CI: 0.79, 1.74 and OR = 1.01; 95% CI: 0.88, 1.16, respectively) and in women compared with men (OR = 1.07; 95% CI: 0.91, 1.26 and OR = 1.00; 95% CI: 0.63, 1.59, respectively), whereas associations were similar for age < 50 and ≥ 50 years.

In the bi-pollutant model, the NO_2_ estimate increased from 1.10 (95% CI: 0.99, 1.21) to 1.17 (95% CI: 0.99, 1.38), whereas the PM_10_ estimate decreased from 1.04 (95% CI: 0.88, 1.23) to 0.98 (95% CI: 0.79, 1.21) (Table 3).

The comparison of ECRHS results using ESCAPE NO_2_ estimates or the previously published APMoSPHERE NO_2_ estimates (Jacquemin et al. 2009b) showed that the effect estimates were sensitive to both the analytic approach and the exposure models. Higher effect estimates were observed in the model with study/city used as fixed effect and/or when using the APMoSPHERE exposure model (see Supplemental Material, Table S6). For instance, the estimate based on the ESCAPE model and random effect on city was 1.04 (95% CI: 0.91, 1.20) and increased up to 1.94 (95% CI: 1.27, 2.96) in the model using the APMoSPHERE air pollution exposure and adjusted on city.

## Discussion

In this longitudinal investigation, asthma incidence was positively associated with all exposure metrics, except with the coarse fraction of PM. The association was borderline statistically significant for a 10-μg/m^3^ increase in NO_2_ (OR = 1.10; 95% CI: 0.99, 1.21) and significant with back-extrapolated NO_2_ (OR = 1.10; 95% CI: 1.00, 1.20). Overall, these findings provide suggestive but not firm evidence for a role of ambient air pollution on asthma incidence in adults.

The main strengths of this study are a large population from a wide geographical area, including > 23,000 participants from eight countries and > 20 different cities across Europe using standardized air pollution estimates at the residential address for a variety of air pollutant metrics. This was achieved through a standardized procedure regarding air pollutants measurements, development of land use regression models, and validation (Beelen et al. 2013; Eeftens et al. 2012). The lack of highly significant associations in our findings is in line with three interpretations: namely, that there is no such association, that pollutants affect only subgroups of adults, or that we were unable to reliably capture such association as a result of epidemiological bias or lack of power. Overall the validity of those LUR models, assessed with the *R*^2^ (see Supplemental Material, Table S5), were good, although this varied across study sites. We showed that restricting the NO_2_ analyses to the centers with higher *R*^2^ did not modify the results. A simulation study showed that LUR modeling with a small number of measurement sites may bias the health-effect estimates in the form of attenuation toward the null (Basagaña et al. 2013). The lack of association with PM may result partly from the small number of measurement sites for these pollutants. A further limitation was the long lag between the health assessments of most of our cohorts and the standardized ESCAPE measurement campaigns, reaching up to 20 years in some of the cohorts. The resulting exposure misclassification likely contributed to imprecise risk estimates and a bias toward the null (Basagaña et al. 2013). To investigate this, back-extrapolated exposure estimates to the follow-up periods for NO_2_ were analyzed. The odds ratio using back-extrapolated values then reached formal statistical significance, but the effect size, which relied mainly on within-city contrasts, was virtually identical to that in the initial analysis. The validity of back-extrapolation of LUR models is supported by a study showing a good correlation between the 1991 back-extrapolated NO_2_ concentrations estimated from the 2009 LUR model and the NO_2_ concentrations measured by monitoring sites in 1991 (Gulliver et al. 2013). However, back-extrapolated exposure estimates will not account for potential changes over time in spatial contrasts within cities, so their validity may vary by location and time. This is an inherent limitation of the ESCAPE project. Nevertheless, associations with other outcomes investigated in ESCAPE, including mortality (Beelen et al. 2014) and lung cancer incidence (Raaschou-Nielsen et al. 2013), have been similar for exposures based on ESCAPE measurement period estimates and exposures based on back-extrapolated estimates.

Caution is necessary when interpreting our findings. Although positive, associations with PM and traffic proximity were nonsignificant, which may indicate that these pollutants do not affect adult-onset asthma or that the analyses lacked statistical power to reliably estimate small effects among rather heterogeneous cohorts. The fact that the positive associations with NO_2_ were the closest to statistical significance does not necessarily mean that NO_2_ is the causal pollutant. It could reflect that our exposure model more accurately estimates the true exposure for this pollutant [which is supported by a trend for a higher *R*^2^ cross-validation of the LUR model for NO_2_ compared with PM_10_ (Beelen et al. 2013; Eeftens et al. 2012)]. Further, given the correlation between pollutant concentrations, we cannot estimate associations with individual pollutants that account for potential confounding by other pollutants. Moreover, no matter how good the exposure models are, there will always be limitations and potential bias in estimating association using exposure estimates only at home addresses that do not account for the individual spatiotemporal activity.

The design induces some limitation regarding the generalizability of our result to other European cities. For all cohorts, the first inclusion criterion was the availability of ESCAPE models, which varied from 20% for E3N (a national study) to 100% for SALIA and NSHD. At the whole cohort level, follow-up rates were less variable, varying between 60% and 80%, which represents a reasonable follow-up rate for such long-term studies (Ackermann-Liebrich et al. 2005; Antó et al. 2010; Kuh et al. 2011; Sanchez et al. 2013; Schikowski et al. 2010; Siroux et al. 2009). Though our study is the largest ever conducted in Europe, with the greatest number of countries and areas, and our estimates did not indicate strong heterogeneity in associations across cohorts, some caution is needed in extrapolating our results, particularly in relation to the heterogeneity between areas, and more importantly to the small sample size in each area.

Defining asthma incidence is more challenging than defining outcomes such as mortality (Beelen et al. 2014) or lung cancer (Raaschou-Nielsen et al. 2013). Furthermore, because adults may not remember early-life wheezing, assessment of adult-onset asthma is difficult (Strachan et al. 1996). A thorough comparison of questionnaires and protocols was undertaken to harmonize asthma definition across the various cohorts without losing valuable information. Although only ECRHS and SAPALDIA were purposefully designed to assess asthma incidence, we were as rigorous as possible in identifying only incident cases, by excluding participants who reported asthma or, when available, asthma-like symptoms at baseline from our study population. Bias in asthma diagnosis may have been introduced through both different cultural perceptions of asthma in the countries in which the cohorts were located, and the different questionnaires and diagnostic protocols used. In the largest cohort included, E3N, the validity of the simple asthma question used has been investigated in a subsample study, which showed good concordance with questions similar to those used in respiratory surveys and with dispensed asthma drug treatment (Sanchez et al. 2013). Because of a limited number of cohorts with bronchial challenge tests, we were unable to perform a sensitivity analysis defining asthma as new bronchial hyperresponsiveness plus symptoms, as used in a previous study of occupational risk factors for asthma (Kogevinas et al. 2007). However, for the three cohorts with information on bronchial hyperresponsiveness, the validity of our incident asthma classification was supported by the increase in bronchial hyperresponsiveness between baseline and follow-up among participants who developed asthma after the baseline examination.

Results should be interpreted in the context of current knowledge and research regarding asthma phenotypes. It is established that childhood-onset asthma, compared with adult-onset asthma, occurs more in males, is more often associated with allergic sensitization, and also depends on specific genetic determinants (Bouzigon et al. 2008; Wenzel 2012). With the increase of childhood asthma, the potential recurrence of asthma in adulthood after remission becomes an increasing concern. Recent research on asthma temporal patterns and data-driven phenotyping conducted in four of the six cohorts included in the present analysis show the complexity of asthma variability over periods of around 10 years in adulthood (Boudier et al. 2013; Sanchez et al. 2013). Asthma in childhood only, adulthood only, old age only, mild (often forgotten) childhood asthma reappearing in adulthood, or persistent asthma throughout the life span are various phenotypes that may depend on both genetic and environmental determinants of various critical windows of expression/exposure. The variability of asthma can be characterized according to different windows of time (Frey and Suki 2008). These may be short (hours or days), often in relation to triggers of attacks, as well as long (months or years). Lessons from other environmental factors (smoking, occupation) have already shown effects on asthma through acute or subchronic exposures. For example, there is increasing evidence of the role of occupational exposure in the various forms of work-related asthma, which encompasses both occupational asthma starting in adulthood and work-exacerbated asthma (Henneberger et al. 2011). The role of occupational exposure has clearly been evidenced in adult-onset asthma assessed in a birth cohort that was followed until adulthood (Ghosh et al. 2013). The follow-up of the numerous birth cohorts initiated in the 1990s and still followed will likely help us understand the various evolutions of the disease.

Our study considered multiple cohorts across Europe, which increased statistical power. However, this also gave potential for larger population heterogeneity, increasing the potential for confounding and therefore bias in the effect estimates. Particular characteristics of each cohort may have influenced the results, such as the health consciousness and high education of the women in E3N or the greater baseline risk of asthma for members of asthmatic families in EGEA. Indeed, as shown in Figure 2, associations were usually largest in EGEA, reaching statistical significance for NO_2_—although this finding was not robust to the exclusion of the 5% most extreme exposure values (data not shown). To investigate cohort-specific influences on results, we formally tested heterogeneity among cohorts and also looked at the robustness of the findings by removing each cohort in turn, which showed some modest variation.

Overall, nearly all the sensitivity and stratified analyses led to ORs > 1. Results from stratified analyses should be interpreted with caution because of the limited number of incident cases in subgroups in some cohorts, and none of the *p*-values for interaction were significant (*p* > 0.35). Surprisingly, the estimates tended to decrease when restricting the analysis to nonmovers for NO_2_ but not for PM_10_. This could be attributable to the lower percentage of movers in E3N and the lack of standardization of moving assessment.

Our results were sensitive to the statistical approach chosen to account for the clustered data, namely using fixed versus random effects for study city/area. Which of the two modeling approaches provides more valid results is difficult to determine, but one factor may be the nature of the air pollutant variation in regards to the within- versus between-city/area. Fixed city/area–effect models estimate purely within-city/area air pollution effects, whereas random-effects models estimate a weighted average of between- and within-city/area effects (Neuhaus and Kalbfleisch 1998). The difference between both approaches within our analyses was driven by the ECRHS estimates, possibly explained by the higher between-city/area variation in air pollutant concentration in this European cohort. Further analyses, including simulation studies, are warranted to better address this statistical issue in the context of the air pollution effect estimates.

Compared with other published results for NO_2_, our confidence intervals largely overlapped those from other studies [OR for 10 μg/m^3^ of NO_2_ = 1.10 (95% CI: 0.99, 1.20) compared with 1.54 (95% CI: 1.00, 2.36) in RHINE (Modig et al. 2009) and 1.43 (95% CI: 1.02, 2.01) in ECRHS (Jacquemin et al. 2009b) and OR for 5.8 ppb of NO_2_ (i.e., 11 μg/m^3^) = 1.12 (95% CI: 0.96, 1.30) in a cohort of U.S. women (Young et al. 2014)]. Interestingly, the association with NO_2_ tended to increase when controlling for PM_10_ concentration. Two of the six cohorts included in our analyses had previously assessed associations between air pollution and asthma incidence in adults. In ECRHS, a positive and significant association was found between individually assigned air pollution exposure derived from a 1 × 1 km air pollution map (APMoSPHERE) and asthma incidence defined in a similar way to ESCAPE (Jacquemin et al. 2009b) and also in an alternative way based on asthma symptoms (Jacquemin et al. 2009a). One possible reason for seeing consistently stronger associations with APMoSPHERE based analyses is that APMoSPHERE used air pollution data closer in time to the collection of health data. Alternatively, a spatially less resolved model may better account for the time activity patterns in adult populations; thus, “background” air pollutant exposure estimates could be a better proxy of the mean individual exposure compared with the very local exposure estimates at the home address, produced by the ESCAPE modeling strategy. SAPALDIA (Künzli et al. 2009) reported significant associations between asthma incidence in never-smokers and individually assigned changes in a specifically modeled marker termed “traffic related PM_10_.” ESCAPE had no such marker, so direct comparisons cannot be made. Moreover, only three SAPALDIA areas were included in ESCAPE—and only one with PM—whereas all previous SAPALDIA results were based on the eight areas the cohort had been designed for in 1990.

Various mechanisms have been proposed to explain the associations of air pollution with asthma. Active ongoing research is being conducted to disentangle the various asthma phenotypes and assess which mechanisms may be specifically involved. Because childhood-onset asthma is more often associated with allergic sensitization, it could be hypothesized that allergy-related mechanisms influence childhood asthma relapsing in adulthood. However, recent results from ESCAPE in children up to 10 years of age did not show evidence of associations of air pollution exposure with allergic sensitization (Gruzieva et al. 2014). This suggests that nonallergic mechanisms, for which interest is increasing for asthma at any age, are particularly important to consider. Increased frailty of the epithelial barrier, inflammation, oxidative stress, and interaction with genetic and epigenetic determinants have been proposed. Research in adults, including subjects from the cohorts included in our analysis, has suggested a role of air pollution in local inflammation measured in exhaled breath condensate and induced sputum (using ESCAPE exposure estimates) (Vossoughi et al. 2014), interaction with oxidative stress genes (Castro-Giner et al. 2009), or novel DNA methylation markers (Sofer et al. 2013). Ambitious programs with comprehensive environmental exposure assessment and biological markers are starting in childhood populations (Vrijheid et al. 2014). Altogether, adult-onset asthma is only one of the various asthma phenotypes, and comprehensive life course approaches should be developed at the environmental and phenotypic levels.

## Conclusion

With > 23,000 adults across Europe followed for 10 years, including 1,257 incident cases of asthma, this is the largest study to estimate the association between traffic-related air pollution, assessed using a standardized and validated method at the individual level, and asthma incidence in adults. Our findings provide suggestive but no firm evidence for a role of air pollution exposure on asthma incidence in adults. Further research with improved individual-level exposure assessment (taking into account, for example, time–activity patterns) and phenotypic characterization in a life-course perspective is needed to better understand the effect of air pollutants on asthma.

## Supplemental Material

(1.2 MB) PDF
