# Supplementary material for: Ambient Air Pollution and Adult Asthma Incidence in Six European Cohorts (ESCAPE)
Source: Environ Health Perspect. 2015 Feb 24;123(6):613–21. doi: 10.1289/ehp.1408206 (PMC4455584; doi:10.1289/ehp.1408206)
Supplement: (1.2 MB) PDF [file ehp.1408206.s001.acco.pdf]

Environ Health Perspect
doi: 10.1289/ehp.1408206

Note to Readers: *EHP* strives to ensure that all journal content is accessible to all readers. However, some figures and Supplemental Material published in *EHP* articles may not conform to 508 standards due to the complexity of the information being presented. If you need assistance accessing journal content, please contact ehp508@niehs.nih.gov. Our staff will work with you to assess and meet your accessibility needs within 3 working days.

Supplemental Material

Ambient Air Pollution and Adult Asthma Incidence in Six European Cohorts (ESCAPE)

Bénédicte Jacquemin, Valérie Siroux, Margaux Sanchez, Anne-Elie Carsin, Tamara Schikowski, Martin Adam, Valeria Bellisario, Anna Buschka, Roberto Bono, Bert Brunekreef, Yutong Cai, Marta Cirach, Françoise Clavel-Chapelon, Christophe Declercq, Roberto de Marco, Audrey de Nazelle, Regina E. Ducret-Stich, Virginia Valeria Ferretti, Margaret W. Gerbase, Rebecca Hardy, Joachim Heinrich, Christer Janson, Deborah Jarvis, Zaina Al Kanaani, Dirk Keidel, Diana Kuh, Nicole Le Moual, Mark J. Nieuwenhuijsen, Alessandro Marcon, Lars Modig, Isabelle Pin, Thierry Rochat, Christian Schindler, Dorothea Sugiri, Morgane Stempfelet, Sofia Temam, Ming-Yi Tsai, Raphaëlle Varraso, Danielle Vienneau, Andrea Vierkötter, Anna L. Hansell, Ursula Krämer, Nicole M. Probst-Hensch, Jordi Sunyer, Nino Künzli, and Francine Kauffmann

Funding and Acknowledgments

ECRHS was supported by the European Commission, as part of their Quality of Life program.

The coordination of ECRHS II was supported by the European Commission, as part of their Quality of Life program. The following bodies funded the local studies in ECRHS II in this article:

Albacete-Fondo de Investigaciones Santarias (grant code: 97/0035-01, 13 99/0034-01, and 99/0034-02), Hospital Universitario de Albacete, Consejería de Sanidad. **Antwerp-**FWO (Fund for Scientific Research)- Flanders Belgium (grant code: G.0402.00), University of Antwerp, Flemish Health Ministry. **Barcelona-**Fondo de Investigaciones Sanitarias (grant code: 99/0034-01, and 99/0034-02), Red Respira (RTIC 03/11 ISC IIF). Ciber of Epidemiology and Public Health has been established and founded by Instituto de Salud Carlos III. **Erfurt**-GSF–National Research Centre for Environment & Health, Deutsche Forschungsgemeinschaft (DFG) (grant code FR 1526/1-1). **Galdakao**-Basque Health Department. **Grenoble**-Programme Hospitalier de Recherche Clinique-DRC de Grenoble 2000 no.2610, Ministry of Health, Direction de la Recherche Clinique, Ministère de l'Emploi et de la Solidarité, Direction Générale de la Santè, CHU de Grenoble, Comité des Maladies Respiratoires de l'Isère. **Ipswich and Norwich**-National Asthma Campaign (UK). **Huelva**-Fondo de Investigaciones Sanitarias (FIS) (grant code: 97/0035-01, 99/0034-01, and 99/0034-02). **Oviedo**-Fondo de Investigaciones Santarias (FIS) (grant code: 97/0035-01, 99/0034-01, and 99/0034-02). **Paris**-Ministère de l'Emploi et de la Solidarité, Direction Générale de la Santé, UCBPharma (France), Aventis (France), Glaxo France, Programme Hospitalier de Recherche Clinique-DRC de Grenoble 2000 no. 2610, Ministry of Health, Direction de la Recherche Clinique, CHU de Grenoble. **Pavia**-Glaxo, Smith & Kline Italy, Italian Ministry of University and Scientific and Technological Research

(MURST), Local University Funding for Research 1998 & 1999 (Pavia, Italy). **Turin**-ASL 4 Regione Piemonte (Italy), AO CTO/ICORMA Regione Piemonte (Italy), Ministero dell'Università e della Ricerca Scientifica (Italy), Glaxo Wellcome spa (Verona, Italy). **Umeå**-Swedish Heart Lung Foundation, Swedish Foundation for Health Care Sciences & Allergy Research, Swedish Asthma & Allergy Foundation, Swedish Cancer & Allergy Foundation. **Verona**-University of Verona; Italian Ministry of University and Scientific and Technological Research (MURST); Glaxo, Smith & Kline Italy.

EGEA is funded in part by PHRC-Paris, PHRC-Grenoble, ANR 05-SEST-020-02/05-9-97, ANR-06-CEBS, ANR-CES-2009, Région Nord Pas-de-Calais, Merck Sharp & Dohme (MSD)

E3N cohort is being studied with the financial support of the Mutuelle générale de l'éducation nationale, the European Community, the Ligue nationale contre le cancer, the Institut Gustave–Roussy, INSERM and a grant from the World Cancer Research Fund (grant no. 2007/14). The specific respiratory health survey in the E3N study was supported by the Fonds de dotation "Recherche en Santé Respiratoire".

NSHD and Professors Hardy and Kuh are supported by core funding and grant funding (U1200632239 and U12309272) from the UK Medical Research Council.

SALIA received funds from the German state (NRW) and federal Ministries of the Environment. The follow-up investigation was funded by the DGUV (German statutory accident assurance) VT 266.1

SAPALDIA received funds from the The Swiss National Science Foundation (grants no 33CSCO-134276/1, 33CSCO-108796, 3247BO-104283, 3247BO-104288, 3247BO-104284,

3247-065896, 3100-059302, 3200-052720, 3200-042532, 4026-028099), the Federal Office for Forest, Environment and Landscape and several Federal and Cantonal authorities.

Measurements and models for PM in Grenoble (ECRHS, EGEA and E3N) were funded by Région Rhône-Alpes.

We also thank all study members and staff involved in data collections in each cohort:

ECRHS

The ECRHS data incorporated in this analysis would not have been available without the collaboration of the following individuals and their research teams.

ECRHS Co-ordinating centre: P Burney, D Jarvis, S Chinn, J Knox (ECRHS II), C Luczynska[+], J Potts.

Steering Committee for ECRHS II: P Burney, D Jarvis, S Chinn, J.M Anto, I.Cerveri, R.deMarco , T.Gislason, J.Heinrich, C. Janson, N. Kunzli, B. Leynaert, F. Neukirch, T. Rochat, J. Schouten, J. Sunyer; C. Svanes, P. Vermeire[+], M. Wjst.

Principal Investigators and Senior Scientific Teams for ECRHS II: Australia: Melbourne (M Abramson, R Woods, EH Walters, F Thien), Belgium: South Antwerp & Antwerp City (P Vermeire[+], J Weyler, M Van Sprundel, V Nelen), Denmark: Aarhus (EJ Jensen), Estonia: Tartu (R Jogi, A Soon), France: Paris (F Neukirch, B Leynaert, R Liard, M Zureik), Grenoble (I Pin, J Ferran-Quentin), Bordeaux (A Taytard, C Raherison), Montpellier (J Bousquet, P Demoly)Germany: Erfurt (J Heinrich, M Wjst, C Frye, I Meyer) Hamburg (K Richter),Iceland: Reykjavik (T Gislason, E Bjornsson, D Gislason, T Blondal, A Karlsdottir), Italy: Turin (M Bugiani, R Bono, P Piccioni, E Caria, A Carosso, E Migliore, G Castiglioni), Verona (R de

Marco, G Verlato, E Zanolin, S Accordini, A Poli, V Lo Cascio, M Ferrari), Pavia (A Marinoni, S Villani, M Ponzio, F Frigerio, M Comelli, M Grassi, I Cerveri, A Corsico),Netherlands: Groningen & Geleen (J Schouten, M Kerkhof), Norway: Bergen (A Gulsvik, E Omenaas, C Svanes, B Laerum), Spain: Barcelona (JM Anto, J Sunyer, M Kogevinas, JP Zock, X Basagana, A Jaen, F Burgos), Huelva (J Maldonado, A Pereira, JL Sanchez), Albacete (J Martinez-Moratalla Rovira, E Almar), Galdakao (N Muniozguren, I Urritia), Oviedo (F Payo), Sweden: Uppsala (C Janson, G Boman, D Norback, M Gunnbjornsdottir), Goteborg (K Toren, L Lillienberg, AC Olin, B Balder, A Pfeifer-Nilsson, R Sundberg), Umea (E Norrman, M Soderberg, K Franklin, B Lundback, B Forsberg, L Nystrom),Switzerland: Basel (N Kunzli, B Dibbert, M Hazenkamp, M Brutsche, U Ackermann-Liebrich); UK: Norwich (D Jarvis, B Harrison), Ipswich (D Jarvis, R Hall, D Seaton), USA: Portland (M Osborne, S Buist, W Vollmer, L Johnson)

[+]Deceased

Coordination: V Siroux (epidemiology, PI since 2013); F Demenais (genetics); I Pin (clinical aspects); R Nadif (biology); F Kauffmann (PI 1992-2012). **Respiratory epidemiology**: Inserm U 700, Paris: M Korobaeff (Egea1), F Neukirch (Egea1); Inserm U 707, Paris: I Annesi-Maesano (Egea1-2); Inserm CESP/U 1018, Villejuif: F Kauffmann, N Le Moual, R Nadif, MP Oryszczyn (Egea1-2), R Varraso; Inserm U 823, Grenoble: V Siroux. **Genetics:** Inserm U 393, Paris: J Feingold; Inserm U 946, Paris: E Bouzigon, F Demenais, MH Dizier; CNG, Evry: I Gut (now CNAG, Barcelona, Spain), M Lathrop (now Univ McGill, Montreal, Canada). Clinical centers: Grenoble: I Pin, C Pison; Lyon: D Ecochard (Egea1), F Gormand, Y Pacheco; Marseille: D Charpin (Egea1), D Vervloet (Egea1-2); Montpellier: J Bousquet; Paris Cochin: A Lockhart (Egea1), R Matran (now in Lille); Paris Necker: E Paty (Egea1-2), P Scheinmann (Egea1-2);

Paris-Trousseau: A Grimfeld (Egea1-2), J Just. **Data and quality management**: Inserm ex-U155 (Egea1): J Hochez; Inserm CESP/U 1018, Villejuif: N Le Moual; Inserm ex-U780: C Ravault (Egea1-2); Inserm ex-U794: N Chateigner (Egea1-2); Grenoble: J Quentin-Ferran (Egea1-2).

E3N

We wish to thank all the women from the E3N study. We also thank Lyan Hoang, Maryvonne Niravong and Marie Fangon for invaluable assistance with the implementation of the study.

SALIA

During the last decades a lot of scientists, study nurses and laboratories were involved in conducting the study. As representatives for all these people we would like to thank especially Reinhard Dolgner (MD) for organizing the baseline study and Barbara Schulten as study nurse for her help in organizing the follow-up study. We are most grateful for all the women from the Ruhr area and from Borken who participated in the study during decades.

SAPALDIA

Study directorate: T Rochat (p), NM Probst Hensch (e/g), JM Gaspoz (c), N Künzli (e/exp), C Schindler (s).

Scientific team: JC Barthélémy (c), W Berger (g), R Bettschart (p), A Bircher (a), G Bolognini (p), O Brändli (p), C Brombach (n), M Brutsche (p), L Burdet (p), M Frey (p), U Frey (pd), MW Gerbase (p), D Gold (e/c/p), E de Groot (c), W Karrer (p), R Keller (p), B Knöpfli (p), B Martin (pa), D Miedinger (o), U Neu (exp), L Nicod (p), M Pons (p), F Roche (c), T Rothe (p), E Russi

Table S1. Study characteristics.

Characteristic	ECRHS	EGEA	E3N	NSHD	SALIA	SAPALDIA
Design of the study	**Cohort of young adults from general urban population in Europe** **(**The European Community Respiratory Health Survey II 2002**)**	**Case (asthma)-control and family study of adults in France** (Siroux et al. 2009)	**Cohort of older women members of a national health insurance plan for teachers** **(**Clavel-Chapelon et al. 1997**)**	**British birth cohort of subject born in 1958** (Kuh et al. 2011**)**	**Cohort of old women living in the highly polluted Ruhr area** **(**Schikowski et al. 2010**)**	**Cohort of adults from general population in cities Switzerland** **(**Ackerman-Liebrich et al. 2005**)**
Website	http://www.ecrhs.org	https://egeanet.vjf.inserm.fr/index.php/en/	http://www.e3n.fr	http://www.nshd.mrc.ac.uk	-	http://www.sapaldia.net
Countries	Belgium, France, Germany, Italy, Spain, Sweden, UK	France	France	UK	Germany	Switzerland
Areas for which each study contributes	Albacete, Antwerp, Barcelona, Erfurt, Grenoble, Huelva, Paris, Pavia, Turin, Umea, Verona, Norwich, Ipswich	Grenoble, Paris, Lyon, Marseille	Grenoble, Paris, Lyon, Marseille	England, Wales, Scotland	Ruhr area	Basel, Geneva, Lugano,
Number of subjects included in the NO_x analyses[a]	3802	517	12763	2339	2073	2210
Mean duration of follow-up in years	11	9	15	10[b]	17	11
Number of incident cases during follow-up	145	49	751	94	148	70

[a]See cohort-specific flow charts for more details (Figure S1). [b]In this analysis, baseline makes reference to the 1989 survey, which is the NSHD baseline for our analyses, and follow-up makes reference to the 1999 survey.

Table S2. Variables used to define asthma at baseline and incident asthma (asthma at follow-up among those without asthma at baseline) for each study.

Variable	ECRHS	EGEA	E3N	NSHD	SALIA	SAPALDIA
**Baseline asthma**						
Ever asthma	Ever asthma, asthma diagnosed by a doctor	Asthma family probands, asthma diagnosed by a doctor, ever asthma attacks, ever attacks of breathlessness with wheezing	Ever asthma attacks	Ever asthma	Asthma diagnosed by a doctor, asthma medication	Ever asthma, asthma diagnosed by a doctor, ever breathless while wheezing
"Current asthma" (last 12 months)	Asthma attacks, asthma medication	Same as ECRHS	N/A	N/A	N/A	Same as ECRHS
At least 3 asthma symptoms in the last 12 months	Breathless while wheezing, woken up with a feeling of chest tightness, attack of SOB at rest, attack of SOB after exercise, woken by attack of SOB	Same as ECRHS	N/A	N/A	N/A	Same as ECRHS
**Incident asthma**						
Ever asthma	Ever asthma	Ever asthma attacks, ever attacks of breathlessness with wheezing	Asthma attacks since last questionnaire	Asthma in the last 10 years (i.e. previous (baseline) questionnaire)	Ever asthma, doctor diagnosed asthma	Ever asthma
"Current asthma" (last 12 months)	Asthma attacks, asthma treatment	Same as ECRHS	Asthma attacks	N/A	N/A	Same as ECRHS

SOB: shortness of breath; N/A: not available.

Analyses were limited to participants who were not classified as positive for any of the asthma variables at baseline. Any articipant providing a positive response on ever asthma at follow-up was considered as an incident case, whatever his/her response to current asthma when available. A stricter definition of incident asthma with a coherent age of onset (reported age at onset at follow-up not earlier than 2 years prior to baseline) was also considered in sensitivity analysis (see main text).

Table S3. Distribution of the air pollutants and traffic variables per study.

Study	N	Mean ± sd	min–max	IQR
**NO_2 (μg/m^3)**				
ECRHS	3802	30±16	0–116	20
EGEA	517	28±11	9–99	16
E3N	12763	31±13	8–94	18
NSHD	2339	22± 7	13–69	10
SALIA	2073	29±8	20–70	10
SAPALDIA	2210	27±7	7–56	8
**Back extrapolated NO_2 to baseline (μg/m^3)**				
ECRHS	1216	39±24	6–113	41
EGEA	277	39±14	17–92	20
E3N	0	NA	NA	NA
NSHD	2276	31±9	19–76	11
SALIA	2071	38±12	20–81	15
SAPALDIA	2208	48±11	11–96	12
**Back extrapolated NO_2 to follow-up (μg/m^3)**				
ECRHS	3802	35±18	0–121	24
EGEA	517	32±13	11–101	18
E3N	12763	34±14	10–101	20
NSHD	2339	26±8	15–78	11
SALIA	same as NO_2			
SAPALDIA	2210	31±8	8–64	8
**NOx (μg/m^3)**				
ECRHS	3802	52±31	0–223	36
EGEA	517	47±26	6–245	28
E3N	12763	57±29	3–234	37
NSHD	2339	38±15	20–160	18
SALIA	2073	47±20	24–139	25
SAPALDIA	2210	45±14	4–112	15
**PM_{10} (μg/m^3)**				
ECRHS	1946	26±9	12–55	8
EGEA	324	25±3	17–38	3
E3N	10436	25±4	16–27	5
NSHD	2114	16±2	12–26	2
SALIA	2073	27±2	24–34	2
SAPALDIA	915	23±3	18–32	3
**Back extrapolated PM_{10} to baseline (μg/m^3)**				
ECRHS	0	NA	NA	NA
EGEA	0	NA	NA	NA
E3N	0	NA	NA	NA
NSHD	0	NA	NA	NA
SALIA	2071	49±8	32–66	7
SAPALDIA	915	46±4	34–62	4
**Back extrapolated PM_{10} to follow-up (μg/m^3)**				
ECRHS	1557	28±6	16–47	10
EGEA	157	27±4	18–39	5
E3N	9739	27±4	17–29	5
NSHD	2114	22±3	16–36	3
SALIA	same as PM_{10}			
SAPALDIA	915	38±4	27–53	5

Study	N	Mean ± sd	min–max	IQR
**$PM_{2.5}$ (µg/m^3)**				
ECRHS	1946	16±5	8–34	5
EGEA	324	15±2	10–22	2
E3N	10436	15±2	10–16	2
NSHD	2114	10±1	8–15	1
SALIA	2071	18±1	15–22	2
SAPALDIA	915	17±2	12–23	1
**$PM_{2.5absorbance}$ (10^{-5}*m^{-1})**				
ECRHS	1667	2.0±0.9	0.8–5.2	1.4
EGEA	157	2.1±0.8	0.9–4.7	1.3
E3N	9736	1.8±0.7	0.9–2.2	0.9
NSHD	2114	1.0±0.2	0.8–3.2	0.3
SALIA	2071	1.5±0.4	1.0–3.4	0.4
SAPALDIA	915	1.9±0.4	0.9–3.2	0.5
**PM_{coarse} (µg/m^3)**				
ECRHS	1946	11±5	4–28	4
EGEA	324	9±2	4–17	3
E3N	10436	9±3	4–10	4
NSHD	2114	6±1	6–10	1
SALIA	2071	10±2	2–15	2
SAPALDIA	915	7±1	4–10	2
**Traffic nearest road (veh/d)**				
ECRHS	2606	5578±11687	0–144554	6580
EGEA	517	6325±9858	0–116863	7276
E3N	12763	6609±8101	0–124848	6993
NSHD	2339	1403±4518	500–86146	0
SALIA	2071	1972±4905	500–84606	0
SAPALDIA	2130	3330±5501	0–45800	4015
**Traffic load 100m (million veh x m /day)**				
ECRHS	2635	1.617±4	0–56.5	1.920
EGEA	517	1.407±3	0–41.6	1.874
E3N	12763	1.367±3	0–50.8	1.845
NSHD	2339	0.321±1	0–21.0	0.000
SALIA	2071	0.772±2	0–16.8	0.878
SAPALDIA	2092	0.948±2	0–18.7	1.447

NA: not available

Table S4. Correlation matrix between pollutants by study.

Study	NO_2	NO_2 back extrapolated to follow-up	NOx	PM_{10}	PM_{10} back extrapolated to follow-up	$PM_{2.5}$	$PM_{2.5}$ absorbance	PM coarse	Traffic intensity	Traffic load
**ECRHS**										
NO_2	1.00									
NO_2 back extrapolated to follow-up	0.96*	1.00								
NOx	0.90*	0.86*	1.00							
PM_{10}	0.79*	0.74*	0.69*	1.00						
PM_{10} back extrapolated to follow-up	0.77*	0.66*	0.57*	0.96*	1.00					
$PM_{2.5}$	0.75*	0.75*	0.57*	0.78*	0.75*	1.00				
$PM_{2.5\ absorbance}$	0.86*	0.90*	0.77*	0.72*	0.58*	0.72*	1.00			
PM coarse	0.76*	0.74*	0.71*	0.85*	0.81*	0.60*	0.82*	1.00		
Traffic intensity	0.53*	0.53*	0.42*	0.40*	0.41*	0.55*	0.52*	0.36*	1.00	
Traffic load	0.52*	0.59*	0.57*	0.36*	0.28*	0.48*	0.59*	0.41*	0.50*	1.00
**EGEA**										
NO_2	1.00									
NO_2 back extrapolated to follow-up	0.99*	1.00								
NOx	0.94*	0.92*	1.00							
PM_{10}	0.58*	0.57*	0.58*	1.00						
PM_{10} back extrapolated to follow-up	0.39*	0.39*	0.41*	1.00*	1.00					
$PM_{2.5}$	0.64*	0.63*	0.63*	0.69*	0.72*	1.00				
$PM_{2.5\ absorbance}$	0.82*	0.82*	0.71*	0.18*	0.18*	0.60*	1.00			
PM coarse	0.73*	0.78*	0.60*	0.55*	0.52*	0.51*	0.76*	1.00		
Traffic intensity	0.38*	0.34*	0.44*	0.35*	0.2752*	0.34*	0.36*	0.11	1.00	
Traffic load	0.56*	0.57*	0.55*	0.36*	0.11	0.49*	0.82*	0.52*	0.47*	1.00
**E3N**										
NO_2	1.00									
NO_2 back extrapolated to follow-up	0.99*	1.00								
NOx	0.90*	0.89*	1.00							
PM_{10}	0.53*	0.53*	0.48*	1.00						
PM_{10} back extrapolated to follow-up	0.52*	0.52*	0.47*	1.0000*	1.00					
$PM_{2.5}$	0.60*	0.61*	0.51*	0.75*	0.76*	1.00				
$PM_{2.5\ absorbance}$	0.76*	0.76*	0.60*	0.33*	0.33*	0.65*	1.00			
PM coarse	0.85*	0.86*	0.66*	0.62*	0.63*	0.67*	0.76*	1.00		
Traffic intensity	0.40*	0.38*	0.45*	0.31*	0.30*	0.34*	0.46*	0.34*	1.00	
Traffic load	0.58*	0.58*	0.58*	0.28*	0.26*	0.35*	0.72*	0.57*	0.47*	1.00
**NSHD**										
NO_2	1.00									
NO_2 back extrapolated to follow-up	0.85*	1.00								
NOx	0.93*	0.78*	1.00							
PM_{10}	0.60*	0.49*	0.62*	1.00						
PM_{10} back extrapolated to follow-up	0.49*	0.58*	0.49*	0.79*	1.00					

Study	NO_2	NO_2 back extrapolated to follow-up	NOx	PM_{10}	PM_{10} back extrapolated to follow-up	$PM_{2.5}$	$PM_{2.5}$ absorbance	PM coarse	Traffic intensity	Traffic load
$PM_{2.5}$	0.90*	0.74*	0.90*	0.63*	0.49*	1.00				
$PM_{2.5\ absorbance}$	0.83*	0.71*	0.76*	0.59*	0.48*	0.66*	1.00			
PM coarse	0.22*	0.19*	0.23*	0.68*	0.56*	0.22*	0.33*	1.00		
Traffic intensity	0.07*	0.04*	0.13*	0.17*	0.13*	0.06*	0.18*	0.20*	1.00	
Traffic load	0.25*	0.20*	0.31*	0.29*	0.24*	0.27*	0.33*	0.33*	0.55*	1.00
**SALIA**										
NO_2	1.00									
NOx	0.98*	1.00								
PM_{10}	0.80*	0.79*	1.00							
$PM_{2.5}$	0.81*	0.81*	0.88*	1.00						
$PM_{2.5\ absorbance}$	0.88*	0.85*	0.94*	0.88*	1.00					
PM coarse	0.78*	0.78*	0.83*	0.79*	0.86*	1.00				
Traffic intensity	0.24*	0.25*	0.15*	0.14*	0.25*	0.15*	1.00			
Traffic load	0.43*	0.33*	0.29*	0.25*	0.45*	0.27*	0.48*	1.00		
**SAPALDIA**										
NO_2	1.00									
NO_2 back extrapolated to follow-up	0.94*	1.00								
NOx	0.90*	0.86*	1.00							
PM_{10}	0.83*	0.83*	0.75*	1.00						
PM_{10} back extrapolated to follow-up	0.78*	0.79*	0.71*	0.92*	1.00					
$PM_{2.5}$	0.71*	0.71*	0.67*	0.69*	0.67*	1.00				
$PM_{2.5\ absorbance}$	0.75*	0.75*	0.75*	0.69*	0.67*	0.70*	1.00			
PM coarse	0.85*	0.85*	0.77*	0.81*	0.76*	0.60*	0.78*	1.00		
Traffic intensity	0.16*	0.20*	0.20*	0.26*	0.23*	0.14*	0.07*	0.21*	1.00	
Traffic load	0.23*	0.24*	0.22*	0.25*	0.23*	0.30*	0.17*	0.11*	0.04*	1.00

*$p<0.05$.

Table S5. Goodness of fit of the LUR model for each city, with cities from North to South. Data are adapted from Eeftens et al. (2012) for PM and Beleen et al. (2013) for NOx).

Centre/Area	Study concerned	Measured concentration (µg/m³) Mean (min–max)	R^2 in each city/area	R^2 cross validation (LOOCV)	RMSE (cross validation (µg/m³)	Number of sites used for model development
**NOx**						
Umea, Sweden	ECRHS	18.9 (2.3–95.9)	87%	82%	7.9	40
London/Oxford, UK	ECRHS, NSHD	69.3 (18.8–257.4)	91%	88%	16.2	40
Antwerp, Belgium	ECRHS	51.8 (17.5–130.8)	87%	82%	11.2	80
Ruhr area, Germany	SALIA	60.0 (26.9–135.7)	88%	81%	13.6	40
Erfurt, Germany	ECRHS	28.8 (15.6–61.8)	87%	84%	4.3	39
Paris, France	ECRHS, EGEA, E3N	80.3 (12.7–248.3)	75%	67%	31.6	40
Grenoble, France	ECRHS, EGEA, E3N	48.2 (6.5–116.2)	82%	74%	11.2	40
Lyon, France	EGEA, E3N	61.7 (6.5–199.2)	75%	65%	22.5	40
Marseilles, France	EGEA, E3N	70.1 (11.9–266.1)	53%	39%	31.6	40
Basel, Switzerland	SAPALDIA	53.1 (21.6–95.7)	61%	52%	12	40
Geneva, Switzerland	SAPALDIA	55.9 (22.1–108.6)	81%	73%	9.1	40
Lugano, Switzerland	SAPALDIA	47.8 (21.2–116.4)	87%	82%	7.4	42
Turin, Italy	ECRHS	101.2 (22.8–101.2)	78%	72%	17	40
Pavia, Italy	ECRHS	50.9 (29.5–117.9)	88%	80%	9.6	20
Verona, Italy	ECRHS	91.8 (33.1–284.4)	64%	54%	32.3	40
Barcelona, Spain	ECRHS	101.3 (21.0–236.4)	73%	65%	27.7	40
Albacete, Spain	ECRHS	42.7 (0.6–148.6)	88%	84%	11	38
Huelva, Spain	ECRHS	33.8 (13.3–71.3)	56%	31%	11.5	24
**NO_2**						
Umea, Sweden	ECRHS	9.3 (1.5–35.8)	87%	83%	2.8	40
London/Oxford, UK	ECRHS, NSHD	37.9 (7.3–102.7)	89%	87%	6.6	40
Antwerp, Belgium	ECRHS	30.9 (12.8–61.5)	86%	81%	5.1	80
Ruhr area, Germany	SALIA	33.2 (20.2–58.4)	89%	84%	4.3	40
Erfurt, Germany	ECRHS	18.6 (11.0–33.4)	89%	87%	2.1	39
Paris, France	ECRHS, EGEA, E3N	39.8 (6.9–96.8)	77%	67%	11.6	40
Grenoble, France	ECRHS, EGEA, E3N	27.2 (5.5–53.2)	83%	78%	4.8	40
Lyon, France	EGEA, E3N	35.0 (7.3–88.0)	90%	72%	8.7	40
Marseilles, France	EGEA, E3N	36.1 (10.0–92.8)	59%	46%	10.7	40
Basel, Switzerland	SAPALDIA	31.0 (16.0–47.8)	67%	58%	4.8	40
Geneva, Switzerland	SAPALDIA	29.3 (16.1–51.3)	87%	81%	3.7	40
Lugano, Switzerland	SAPALDIA	28.6 (12.2–59.1)	87%	82%	3.5	42

Centre/Area	Study concerned	Measured concentration (µg/m³) Mean (min–max)	R^2 in each city/area	R^2 cross validation (LOOCV)	RMSE (cross validation (µg/m³)	Number of sites used for model development
Turin, Italy	ECRHS	53.3 (15.6–83.7)	78%	70%	7.7	40
Pavia, Italy	ECRHS	25.9 (15.7–53.4)	92%	87%	3.3	20
Verona, Italy	ECRHS	41.6 (16.3–100.1)	64%	55%	10.8	40
Barcelona, Spain	ECRHS	57.7 (13.8–109.0)	75%	68%	11.6	40
Albacete, Spain	ECRHS	26.1 (1.9–75.5)	90%	87%	5.2	38
Huelva, Spain	ECRHS	21.9 (8.4–43.4)	55%	31%	7	24
**PM_{10}**						
London/Oxford, UK	ECRHS, NSHD	18.6(12.1–31.2)	90%	88%	1.5	20
Antwerp, Belgium	ECRHS	27.1 (21.9–37.0)	68%	60%	2.3	40
Ruhr area, Germany	SALIA	27.9 (22.5–33.6)	69%	63%	2	20
Paris, France	ECRHS, EGEA, E3N	25.6 (16.6–52.4)	87%	77%	3.5	20
Grenoble, France[a]	ECRHS, EGEA, E3N	25.8 (18.2–34.6)	89%	80%	2.1	20
Lugano, Switzerland	SAPALDIA	23.9 (18.5–32.4)	87%	80%	1.6	18
Turin, Italy	ECRHS	43.1 (31.5–57.8)	78%	69%	3.9	20
Barcelona, Spain	ECRHS	37.4 (17.8–48.5)	87%	82%	3.1	20
**$PM_{2.5}$**						
London/Oxford, UK	ECRHS, NSHD	11.2 (7.0–21.1)	82%	77%	1.4	20
Antwerp, Belgium	ECRHS	17.1 (12.7–21.5)	67%	61%	1.2	40
Ruhr area, Germany	SALIA	18.5 (15.5–21.6)	88%	79%	0.9	20
Paris, France	ECRHS, EGEA, E3N	16.0 (11.9–30.6)	89%	73%	1.8	20
Grenoble, France[b]	ECRHS, EGEA, E3N	15.4 (12.2–20.8)	89%	79%	1.1	20
Lugano, Switzerland	SAPALDIA	17.2 (13.7–22.5)	83%	77%	1.1	19
Turin, Italy	ECRHS	29.3 (22.7–36.3)	71%	59%	2	20
Barcelona, Spain	ECRHS	16.3 (8.4–24.4)	83%	71%	2.1	20
**$PM_{2.5absobance}$**						
London/Oxford, UK	ECRHS, NSHD	1.6 (0.9–4.7)	96%	92%	0.2	20
Antwerp, Belgium	ECRHS	1.7 (0.9–3.0)	92%	89%	0.2	40
Ruhr area, Germany	SALIA	1.6 (1.0–2.6)	97%	95%	0.1	20
Paris, France	ECRHS, EGEA, E3N	2.0 (0.8–5.1)	91%	81%	0.4	20
Lugano, Switzerland	SAPALDIA	2.0 (1.2–3.0)	79%	71%	0.3	19
Turin, Italy	ECRHS	3.0 (1.6–4.2)	88%	81%	0.3	20
Barcelona, Spain	ECRHS	2.7 (0.9–4.9)	86%	80%	0.4	20
**PM coarse**						
London/Oxford, UK	ECRHS, NSHD	7.4 (4.4–10.3)	68%	57%	1.3	20
Antwerp, Belgium	ECRHS	9.3 (6.4–15.0)	51%	38%	1.7	40
Ruhr area, Germany	SALIA	9.4 (7.1–12.8)	66%	57%	1.2	20

Centre/Area	Study concerned	Measured concentration ($\mu g/m^3$) Mean (min–max)	R^2 in each city/area	R^2 cross validation (LOOCV)	RMSE (cross validation ($\mu g/m^3$)	Number of sites used for model development
Paris, France	ECRHS, EGEA, E3N	9.6 (3.9–21.8)	81%	73%	4.6	20
Grenoble, France[c]	ECRHS, EGEA, E3N	10.4 (5.3–17.2)	73%	53%	2.1	20
Lugano, Switzerland	SAPALDIA	6.8 (3.8–9.9)	77%	65%	1.1	18
Turin, Italy	ECRHS	13.8 (7.5–21.5)	65%	58%	2.4	20
Barcelona, Spain	ECRHS	21.0 (9.4–26.0)	75%	70%	2.3	20

LOOCV: leave-one-out-cross- validation; RMSE root mean square error

All LUR models data, but PM for Grenoble, were previously published (Beelen et al. 2013, Eeftens et al 2012)

[a]LUR model for PM_{10}: 36.92 + 2.69E-5 × (total heavy-duty traffic load of all roads in a 50m buffer) – 0.71 × (altitude) + 3.64E-2 × (product of heavy-duty traffic intensity on nearest road and inverse of distance to the nearest road) -9.4E-8 × (Semi-natural and forested areas in a 5000m buffer). [b]LUR model for $PM_{2.5}$: 12.02 + 1.91E-2 × (product of heavy-duty traffic intensity on nearest road and inverse of distance to the nearest road) + 7.07E-6 × (total heavy-duty traffic load of all roads in a 100m buffer) + 9.69E-5 × (low density residential land in a 100m buffer) + 8.44E-5 × (industry in a 100m buffer) – 1.05E-6 × (Semi-natural and forested areas in a 1000m buffer). [c]LUR model for PM coarse: 5.7+1.24E-6 × (low density residential land in a 5000m buffer) + 3.20E-3 × (heavy-duty traffic intensity on nearest major road).

Table S6. Comparison of associations between asthma incidence per 10 μg/m^3 increase for NO_2 between ESCAPE and APMoSPHERE estimates in ECRHS.

Statistical model	Air pollution estimate	OR (95%CI)
Basic model	APMoSPHERE	1.11 (0.97,1.28)
Basic model further adjusted by city (fixed effect)	APMoSPHERE	1.94 (1.27,2.95)
Basic model taking into account city as a random effect	APMoSPHERE	1.16 (0.95,1.43)
Basic model	ESCAPE	1.00 (0.89,1.12)
Basic model further adjusted by city (fixed effect)	ESCAPE	1.18 (0.98,1.43)
Basic model taking into account city as a random effect	ESCAPE	1.04 (0.91,1.20)

This table includes the population with both estimates (3,378 participants), and used a basic statistical model (age, sex, maximum education, smoking at baseline).

APMosphERE estimates were developed using GIS-based modelling techniques (Vienneau et al. 2009)

References

Ackermann-Liebrich U, Kuna-Dibbert B, Probst-Hensch NM, Schindler C, Felber Dietrich D, Stutz EZ, et al. 2005. Follow-up of the Swiss cohort study on air pollution and lung diseases in adults (SAPALDIA 2) 1991-2003: Methods and characterization of participants. Soz Praventivmed 50:245-263.

Beelen R, Hoek G, Vienneau D, Eeftens M, Dimakopoulou K, Pedeli X, et al. Development of NO2 and NOx land use regression models for estimating air pollution exposure in 36 study areas in Europe – the ESCAPE project. 2013. Atmos Environ 72:10-23.

Clavel-Chapelon F, van Liere MJ, Giubout C, Niravong MY, Goulard H, Le Corre C, et al. E3N, a French cohort study on cancer risk factors. E3N group. 1997 Etude épidémiologique auprès de femmes de l'éducation nationale. [in French] Eur J Cancer Prev 6:473-478.

Eeftens M, Beelen R, de Hoogh K, Bellander T, Cesaroni G, Cirach M, et al. 2012. Development of land use regression models for PM(2.5), PM(2.5) absorbance, PM(10) and PM(coarse) in 20 European study areas; results of the ESCAPE project. Environ Sci Technol 46:11195-11205.

Kuh D, Pierce M, Adams J, Deanfield J, Ekelund U, Friberg P, et al. 2011. Cohort profile: Updating the cohort profile for the MRC National Survey of Health and Development: A new clinic-based data collection for ageing research. Int J Epidemiol 40:e1-9.

Schikowski T, Ranft U, Sugiri D, Vierkötter A, Bruning T, Harth V, et al. 2010. Decline in air pollution and change in prevalence in respiratory symptoms and chronic obstructive pulmonary disease in elderly women. Respir Res 11:113.

Siroux V, Boudier A, Bousquet J, Bresson JL, Cracowski JL, Ferran J, et al. 2009. Phenotypic determinants of uncontrolled asthma. J Allergy Clin Immunol 124:681-687 e683.

The European Community Respiratory Health Survey II. 2002. The European Community Respiratory Health Survey II. Eur Respir J 20:1071-1079.

Vienneau D, de Hoogh K, Briggs D. 2009. A GIS-based method for modelling air pollution exposures across Europe. Science of the Total Environment 408:255-266.

Figure S1. Flow charts for each study.

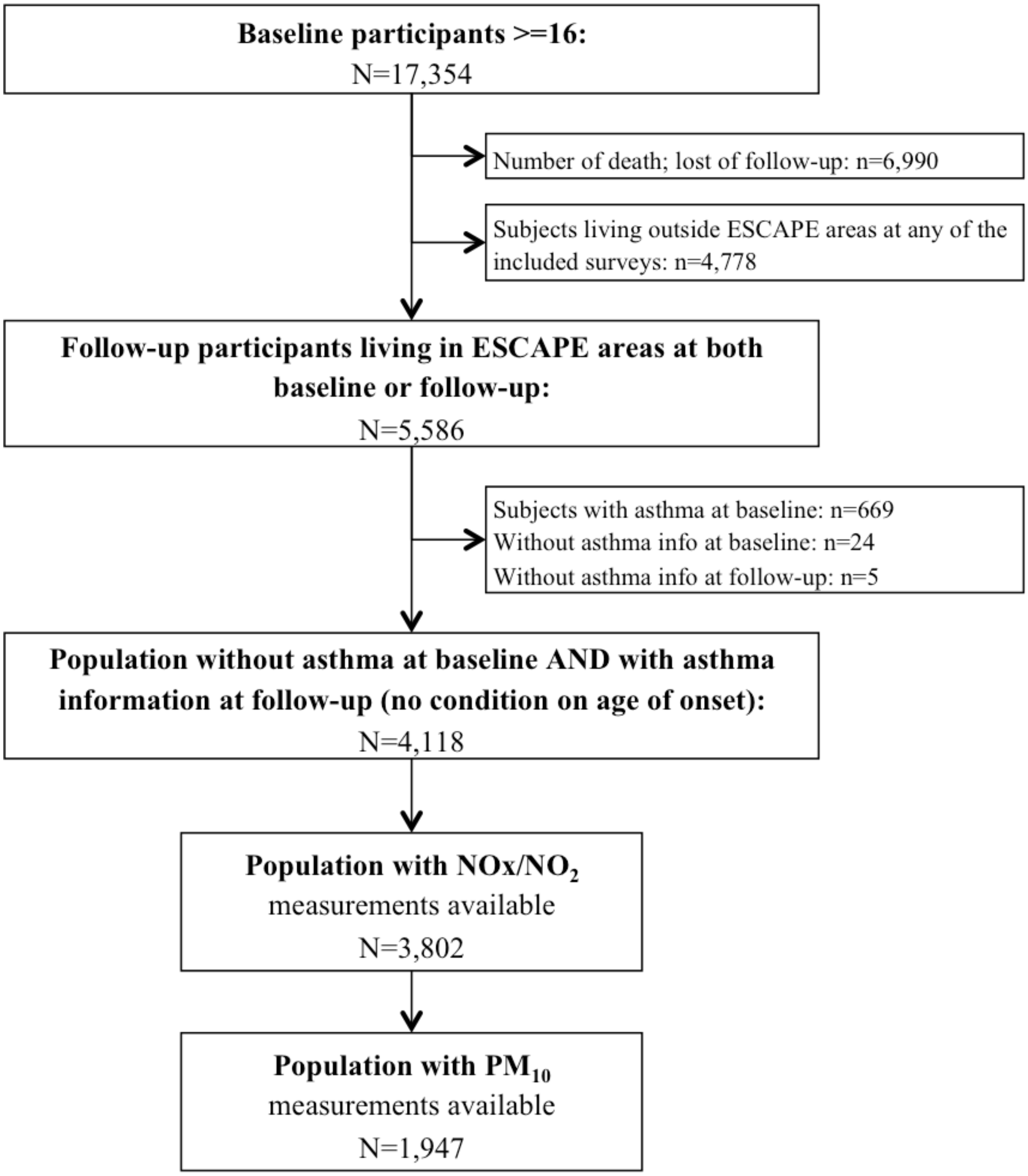

Figure S1a. European Community Respiratory Health Survey (ECHRS). Participants from the enriched (symptomatic) sample are excluded. FU: Follow-up. (The European Community Respiratory Health Survey II 2002).

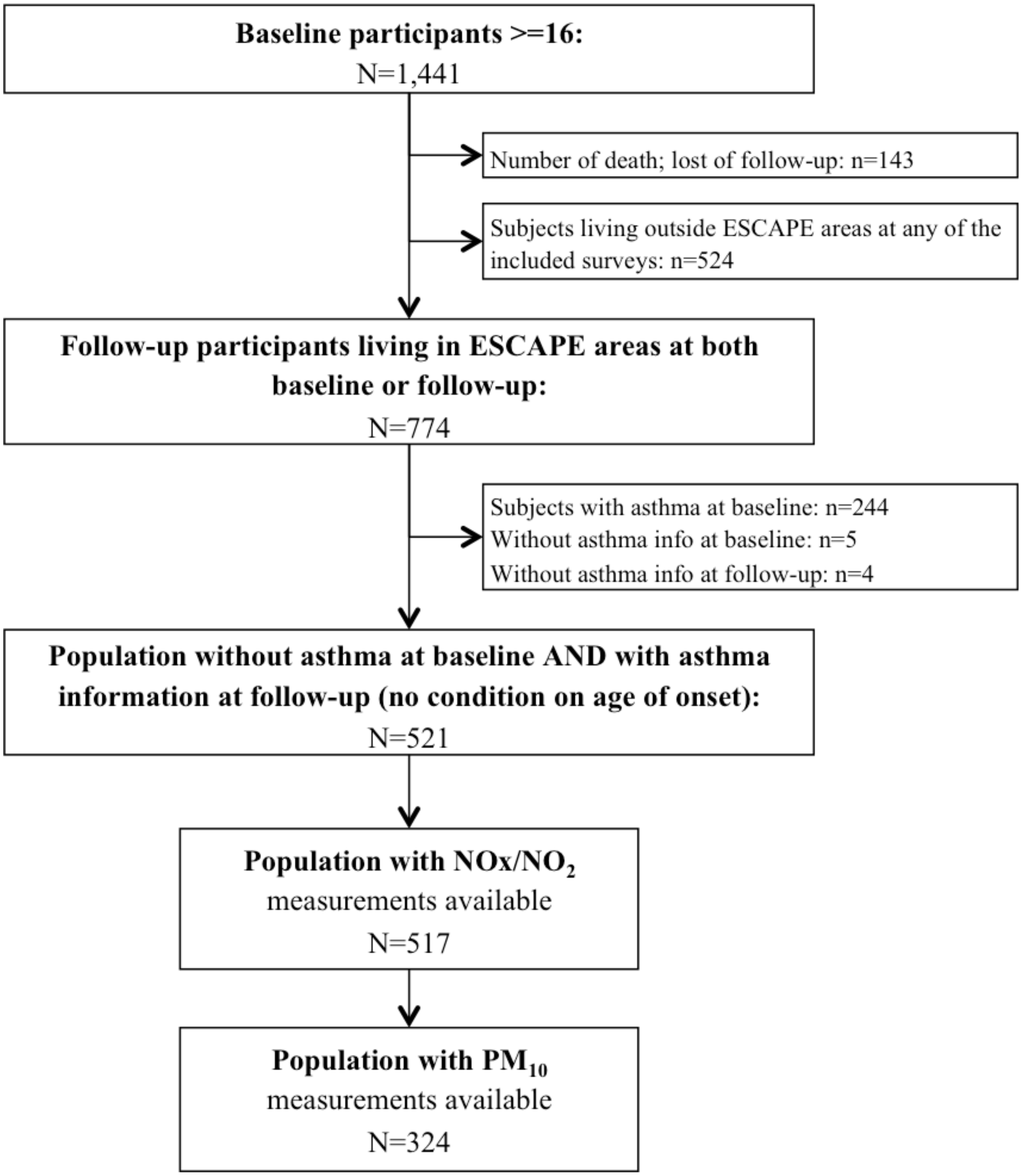

Figure S1b. Epidemiological study on the Genetics and Environment of Asthma (EGEA). Only adults from the study were included. FU: Follow-up (Siroux et al. 2009).

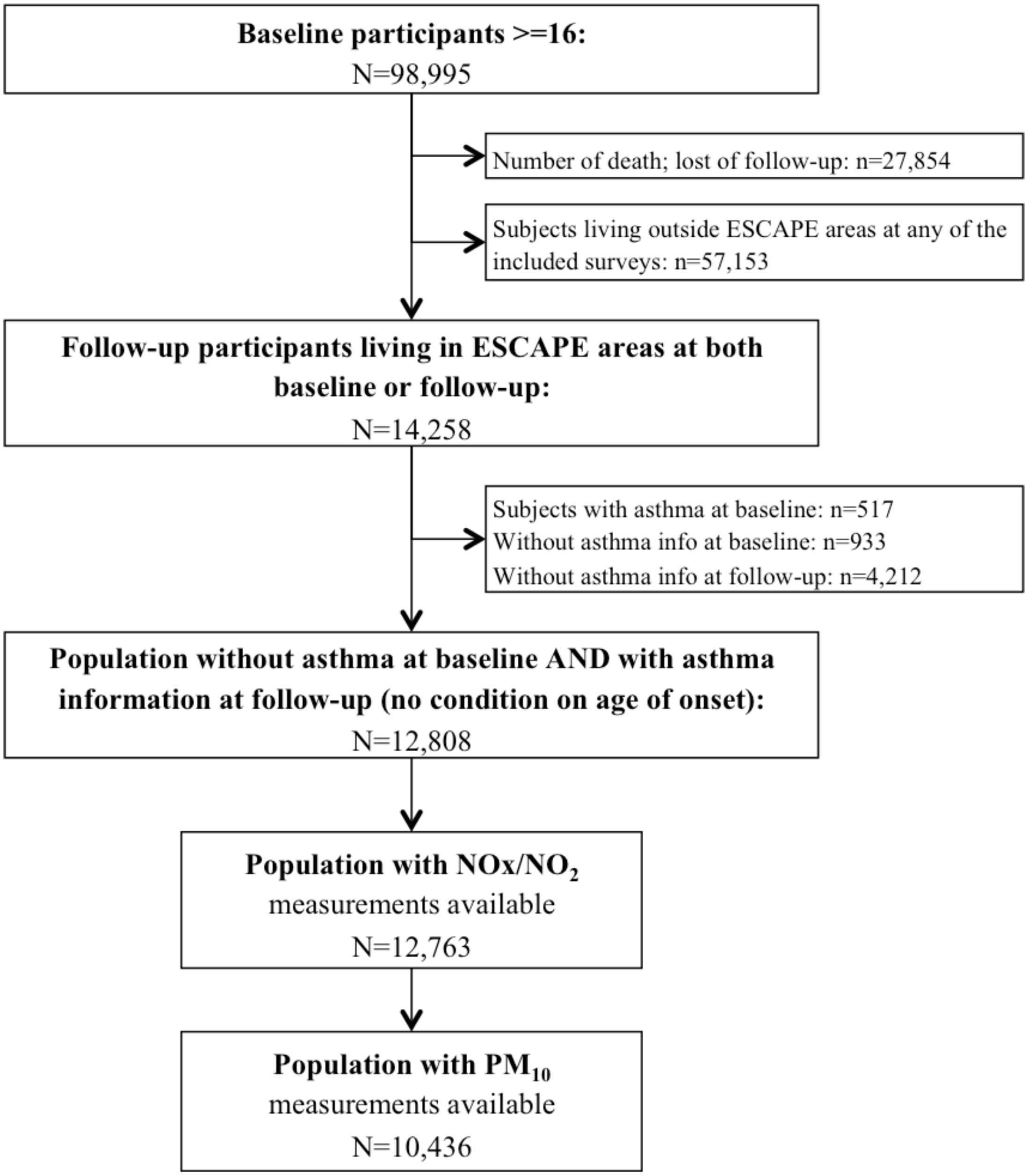

Figure S1c. Etude Epidémiologique auprès de femmes de la Mutuelle Générale de l'Education Nationale (E3N). FU: Follow-up (Clavel-Chapelon et al. 1997).

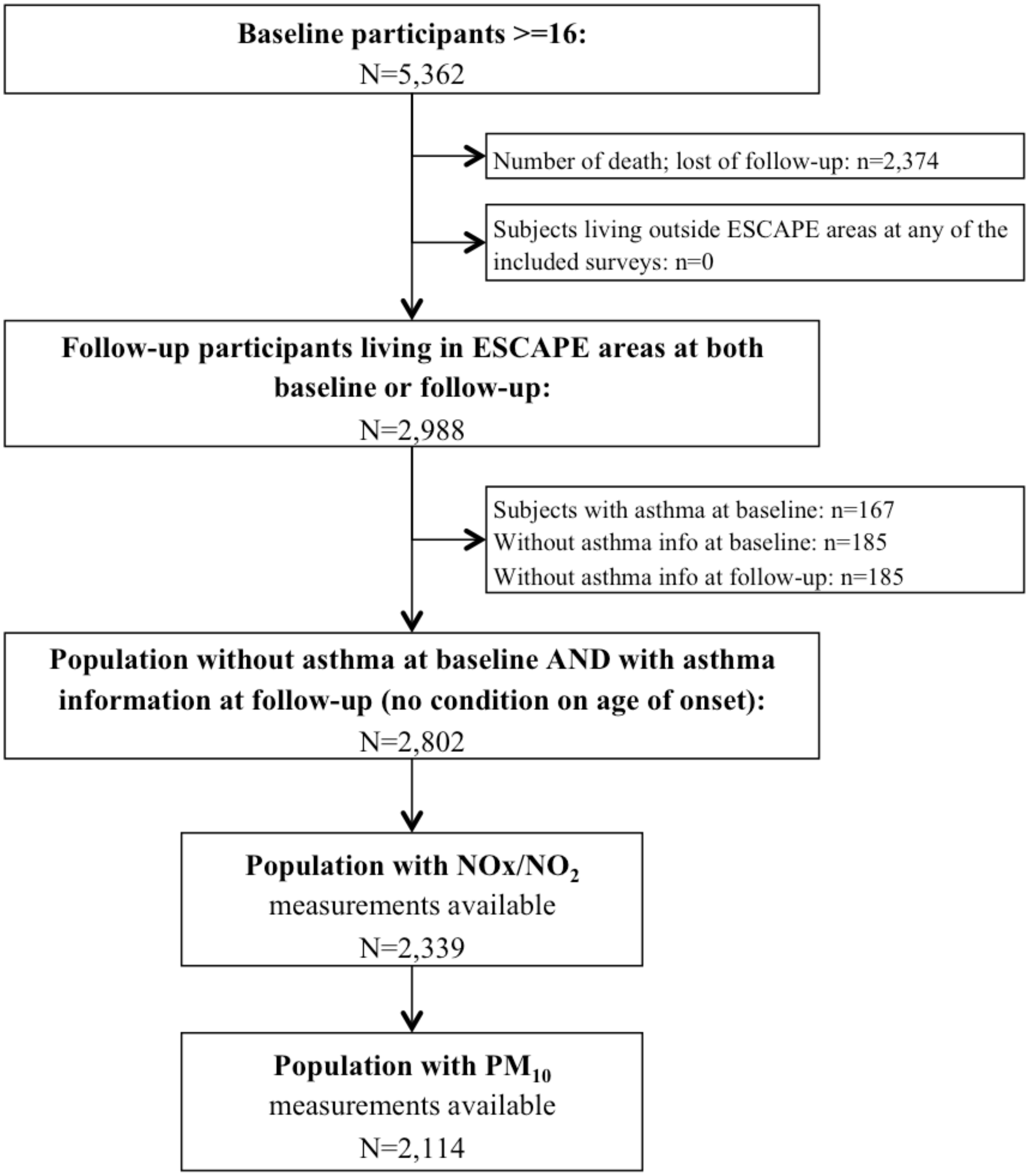

Figure S1d. Medical Research Council's National Survey of Health and Development (NSHD).

Baseline refers for our analyses to the 1989 surveyand FU (follow-up) to the 1999 survey (Kuh et al. 2011)

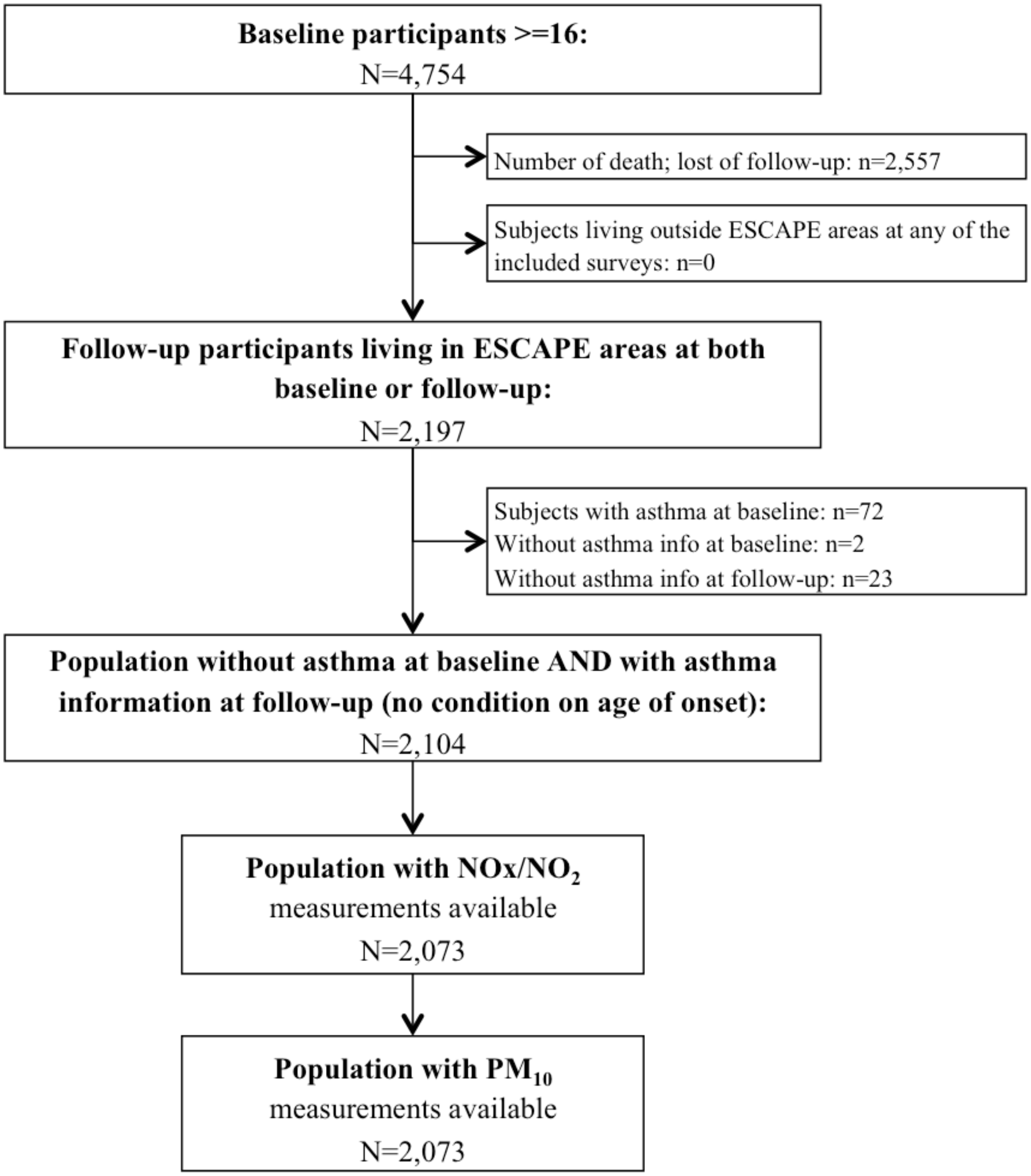

Figure S1e. Study on the influence of Air pollution on Lung function, Inflammation and Aging (SALIA). FU: Follow-up (Schikowski et al. 2010).

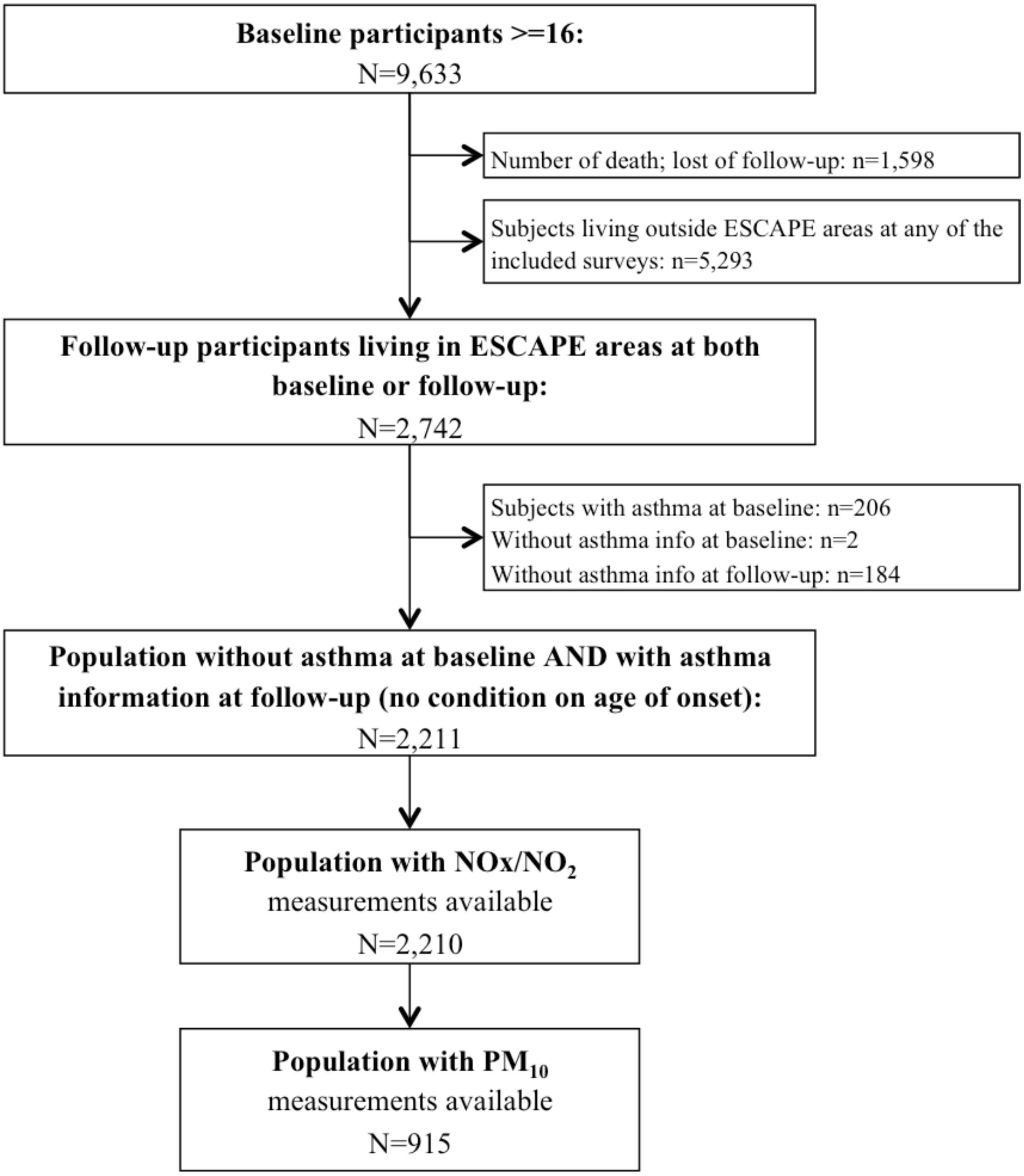

Figure S1f. Swiss study on air pollution and health in adults (SAPALDIA). FU: Follow-up (Ackerman-Liberich et al. 2005).
